# CRISPR-Associated Primase-Polymerases are implicated in prokaryotic CRISPR-Cas adaptation

**DOI:** 10.1038/s41467-021-23535-9

**Published:** 2021-06-17

**Authors:** Katerina Zabrady, Matej Zabrady, Peter Kolesar, Arthur W. H. Li, Aidan J. Doherty

**Affiliations:** 1 Genome Damage and Stability Centre, School of Life Sciences, University of Sussex, Brighton, UK; 2 Present Address: National Centre for Biomolecular Research, Masaryk University, Brno, Czech Republic

**Keywords:** DNA, Enzyme mechanisms, DNA repair enzymes, CRISPR-Cas systems

## Abstract

CRISPR-Cas pathways provide prokaryotes with acquired “immunity” against foreign genetic elements, including phages and plasmids. Although many of the proteins associated with CRISPR-Cas mechanisms are characterized, some requisite enzymes remain elusive. Genetic studies have implicated host DNA polymerases in some CRISPR-Cas systems but CRISPR-specific replicases have not yet been discovered. We have identified and characterised a family of CRISPR-Associated Primase-Polymerases (CAPPs) in a range of prokaryotes that are operonically associated with Cas1 and Cas2. CAPPs belong to the Primase-Polymerase (Prim-Pol) superfamily of replicases that operate in various DNA repair and replication pathways that maintain genome stability. Here, we characterise the DNA synthesis activities of bacterial CAPP homologues from Type IIIA and IIIB CRISPR-Cas systems and establish that they possess a range of replicase activities including DNA priming, polymerisation and strand-displacement. We demonstrate that CAPPs operonically-associated partners, Cas1 and Cas2, form a complex that possesses spacer integration activity. We show that CAPPs physically associate with the Cas proteins to form bespoke CRISPR-Cas complexes. Finally, we propose how CAPPs activities, in conjunction with their partners, may function to undertake key roles in CRISPR-Cas adaptation.

## Abstract (web summary)

CAPPs are putative Primase-Polymerases associated with CRISPR-Cas operons. Here, the authors show CAPPs genetic and physical association with Cas1 and Cas2, their capacity to function as DNA-dependent DNA primases and DNA polymerases, and that Cas1-Cas2 complex adjacent to CAPP has bona fide spacer integration activity.

## Introduction

Cells contain a diverse range of canonical DNA polymerases critical for replication, repair and damage tolerance mechanisms that maintain genome stability. However, cells also contain non-canonical polymerases called primases that historically were considered to play roles limited to primer synthesis. Two distinct superfamilies of primases have been identified, DnaG and archaeo-eukaryotic primases (AEPs)^1,2^. DnaG primases comprise a catalytic domain of the topoisomerase-primase (TOPRIM) fold^3^ and are found in prokaryotic and viral organisms. In contrast, AEP members, originally identified in archaea and eukaryotes but are also widely found in bacteria^4^, are structurally distinct and are found in all domains of life. The classical role of primases is to synthesise short RNA primers that provide the critical 3′-hydroxyl moiety that allows replicative polymerases to elongate primers to copy the cellular DNA. However, in recent years it has become evident that AEP family members display distinct functional diversity and undertake varied functions in DNA metabolism, including roles in replication, repair and damage tolerance^5^.

The recent recognition that AEP members have diversified to undertake functionally distinct roles in cells, from bacteria to mammals, has led to a proposal to reclassify all members of the AEP superfamily into a sub-group of polymerases called Primase-Polymerases (Prim-Pols), a name that more accurately reflects both their evolutionary origins and more diverse functions^5^. This diversification of Prim-Pol functions is exemplified by the Ligase D-associated Prim-Pol (PPD), required for the repair of double-strand breaks (DSBs) in many prokaryotic organisms^5–8^. The closely related bacterial Prim-PolC (PPC) is specifically involved in the repair of DNA gapped intermediates produced during excision repair^9^. A second primase called PrimPol was also identified in eukaryotic cells that acts predominantly as a repriming enzyme that facilitates the restart of stalled DNA replication^10^. The discovery and characterisation of functionally diverse Prim-Pol’s, in both prokaryotic and eukaryotic organisms, with bespoke roles in a variety of DNA metabolic pathways, suggests that other Prim-Pols have likely evolved to undertake additional, yet undescribed, roles in cells. Recently, Prim-Pol families associated with diverse mobile genetic elements and others with predicted roles in RNA repair and silencing pathways were reported^4,11^.

The presence of genes encoding putative Prim-Pols was also noted in a range of Clustered Regularly Interspaced Short Palindromic Repeats (CRISPR)-associated (CRISPR-Cas) operons^5,12^. CRISPR-Cas systems provide acquired immunity that confers resistance to foreign genetic elements arising from phage and plasmids. The mechanism of action of CRISPR–Cas systems can be divided into three stages: adaptation, expression and interference. During the adaptation stage, foreign DNA is cleaved to produce fragments that are bound by Cas1-Cas2 complexes, which acquire these new spacer sequences and subsequently integrates these into CRISPR spacer arrays in the bacterial genome. During the expression stage, the CRISPR array is then transcribed and this precursor RNA is processed into individual CRISPR RNAs (crRNAs) containing a single spacer with repeat sequence and the crRNA is bound effector Cas proteins. During the interference stage, the assembled Cas–crRNA complex uses the complementarity of the crRNA to identify invading nucleic acids, which it then cleaves and degrades^13^. CRISPR-Cas systems are divided into two classes, depending on the number of effector proteins forming the Cas-crRNA complex. Class 1 contains systems with multi-subunit effector complexes (e.g. Cmr, Csm) and class 2 possess single-protein effectors (e.g. Cas9, Cas12). Depending on Cas protein composition and their mode of action, each class can then be separated into several types (class 1 – types I, III, IV; class 2 – types II, V, VI)^14^.

Although CRISPR-Cas systems have been under intense scrutiny in recent years, several aspects of the adaptation stage remain unclear^13^. There are two known types of adaptation stage, primed and naïve, both requiring double-stranded prespacers for integration into CRISPR loci. It is still not fully understood how prespacers are produced from the invading nucleic acids. A study of naïve adaptation in *Escherichia coli* (*E.coli*) suggested that the substrates for integration are degraded DNA intermediates formed by the RecBCD dsDNA break repair complex^15^. However, RecBCD is thought to create ssDNA fragments and Cas1-Cas2 complex can only integrate a double-stranded prespacer^16^. Whether this issue is resolved by reannealing of the nucleolytic fragments or by synthesising the complementary second strand using a DNA primase/polymerase is unknown. The nuclease activity of RecBCD complex is dispensable for *E. coli* spacer adaptation but its helicase activity is essential, which suggests the involvement of alternative nucleases^17^. Some non-Cas exonucleases e.g. DnaQ, EcoT were shown to play a role in in vitro processing of prespacers to make them suitable for integration into a CRISPR array^18,19^. During primed adaptation, new spacer acquisition is dependent on already present spacers originating from the same invading DNA, with the target site partially mutated to escape bacterial immunity^20,21^. This type of adaptation is also dependent on the CRISPR effector complex (e.g. Cascade or CMR)^20^ and helicase-nuclease Cas3 protein in *E. coli* CRISPR type I-E^22^. The RecBCD complex is also involved in the in vivo primed adaptation of type I-E CRISPR-Cas. The activity of this complex in this process was found to be redundant with SbcCD nuclease and their function is upstream of RecJ nuclease, which probably has a role in prespacer processing^23^. However, the general mechanism of production and processing of Cas1-Cas2 prespacers during primed adaptation is still poorly understood. Although the general molecular mechanism of prespacer integration into CRISPR arrays by Cas1-Cas2 has been described, the identity and mechanisms of polymerases involved in spacer adaptation and integration processes remains unclear.

Putative Prim-Pols were identified in a number of CRISPR containing operons^5,12^. This genetic association suggests that CRISPR-associated Prim-Pols (CAPPs) are potential candidates to fulfil requisite synthesis roles in these CRISPR-Cas systems. Here, we present a bioinformatic and functional characterisation of Prim-Pols associated with CRISPR-Cas operons. We reveal their distribution, domain structures and operonic associations. CAPPs possess a broad range of synthesis activities, including the capacity to act as both a DNA-dependent DNA primase and DNA polymerase. We establish a strong genetic association between CAPPs and Cas1-Cas2 genes and show that they physically interact together to form a complex. Critically, a Cas1-Cas2 complex found adjacent to CAPP possesses bona fide spacer integration activity, supporting their proposed role in CRISPR spacer integration. Together, these studies provide characterisation of CRISPR-associated replicases and offers insights into how these enzymes, in collaboration with their operonic partners, may contribute to CRISPR-Cas adaptation processes.

## Results

### CRISPR-associated Prim-Pols in prokaryotic organisms

**Fig. 1:**
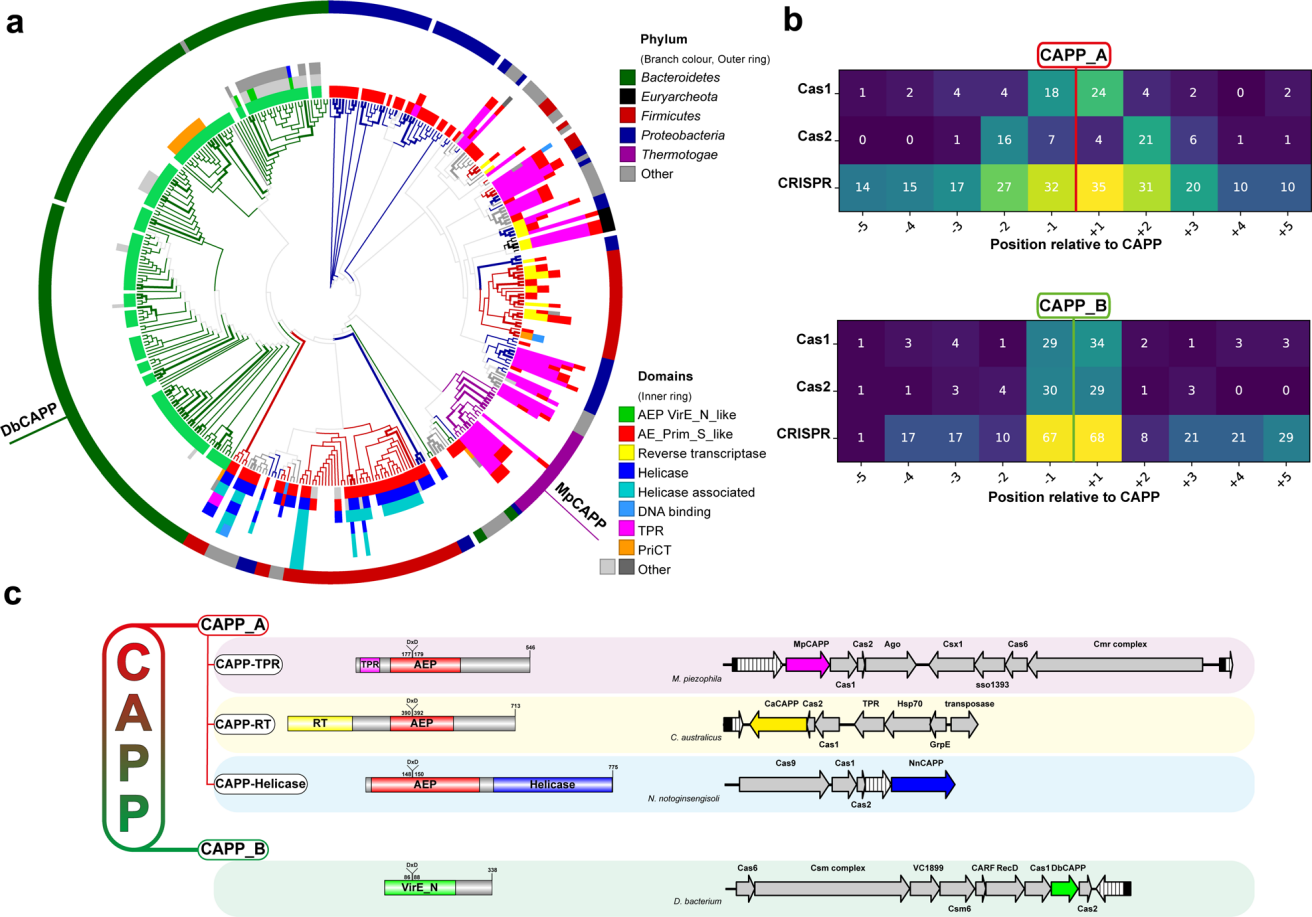
Bioinformatic analysis of CRISPR-associated Prim-Pols. **a** A phylogenetic tree (bootstrap *n* = 100) from multiple sequence alignments of all identified CAPP proteins (Supplementary Data 1 and 2). Branch colours and outer ring colours indicate phyla from the National Center for Biotechnology (NCBI) taxonomy database. The inner ring is composed of coloured protein domain annotations of CAPP proteins from the NCBI Conserved Domains Database (CDD; abbreviated terms are used to simplify colour coding, see Supplementary Fig. 1 for full description). Bootstrap values of 100% are indicated with bold branches. **b** Coloured heatmap of keywords from gene names, upstream and downstream of the *CAPP* genes, indicating their occurrence (%) at certain positions relative to *CAPP*. **c** Classification of CAPP proteins based on their sequence homology, forming two major classes CAPP_A and CAPP_B. CAPP_A is further divided to three types, based on the protein domain architectures, CAPP-TPR, CAPP-RT and CAPP-Helicase. For each class, an example of NCBI CDD derived domain architecture is shown (left), together with its corresponding operonic region (right) showing the neighbouring genes present in each operon. CRISPR repeat array – white textured arrow. TPR – tetratricopeptide repeat – magenta domain, RT – reverse transcriptase – yellow domain, VirE_N – virulent protein E N-terminal like – green domain, helicase - blue domain.

Previous studies identified the presence of putative Prim-Pol-like genes in class 1 type III CRISPR systems in a number of bacterial species^5,12^. We propose that these putative genes/proteins be called CRISPR-associated Prim-Pols (CAPPs) given their operonic association with CRISPR-Cas genes. To identify additional CAPP homologues and further investigate their occurrence, distribution, domain architectures and operonic associations, we performed comprehensive bioinformatics analyses on members of this CRISPR-associated Prim-Pol family, see Methods section. Initial homology searches uncovered two potentially distinctive clades of Prim-Pols, related either to *Thermatogae* or *Bacteroidetes* Prim-Pols. Several iterations of Position Specific Iteration – Basic Local Alignment Search Tool (PSI-BLAST)^24^ searches, using either the sequence of the putative catalytic domain of *Marinitoga sp. 1137* (Msp) (APT75355) or *Dysgonamonadaceae bacterium* (Db) (PLB86576), identified a diverse set of distant homologues. These grouped into two large - non-intersecting – Db (Supplementary Data 1) and Msp (Supplementary Data 2) derived datasets containing bacterial, archaeal and viral Prim-Pols (Fig. 1a). We next searched for upstream and downstream CRISPR genes for every BLAST-derived homologue, to identify Prim-Pols in close proximity of CRISPR-cas genes ascribed in both Genbank^25^ and RefSeq^26^ databases. Analysis of such regions identified a strong genetic association of *CAPP* genes with CRISPR, *Cas1* and *Cas2*, evident in a heatmap of neighbouring genes (Fig. 1b). Full-length sequences of CAPP protein homologues from both, Db- and Msp- datasets were aligned and a phylogenetic tree was built to uncover their sequence relationships, in conjunction with metadata analysis of their corresponding taxonomy and protein domain annotations (Fig. 1a and Supplementary Fig. 1). These analyses identified the presence of two distinct CAPP classes, CAPP_A (Msp-related) and CAPP_B (Db-related) (Supplementary Fig. 1).

CAPP_A class is more abundant and diverse, covering multiple taxonomy branches, including bacterial and archaeal CAPPs. A strong correlation was found between the taxonomy and the protein domain architecture, showing a certain architecture is well confined within certain phyla, suggesting a horizontal gene transfer origin^27^ (Fig. 1a). CAPP_A class can be further divided into three types, based on their protein domain architecture (Fig. 1c). The first type, exemplified by a CAPP (AEX84846) from *Marinitoga piezophila*, can be identified by a number of discernible domains. An N-terminal tetratricopeptide repeat (TPR) domain followed by an archaeo-eukaryotic primase (AEP) catalytic domain and a C-terminal region, potentially containing a PriCT domain (abbreviated as CTD domain). The second type, exemplified by a CAPP (CCJ33120) from *Caloramator australicus*, contains an N-terminal reverse transcriptase domain fusion with a Prim-Pol catalytic domain. The third type, exemplified by a CAPP (RHW43474) from *Neobacillus notoginsengisoli*, contains a Prim-Pol catalytic domain followed by a helicase domain.

CAPP_B class consists of proteins with a simpler domain architecture, lacking other readily discernible domains fused to an AEP catalytic domain. Notably, these catalytic domains are ascribed as VirE N-terminal domains, typically fused to virulence-associated protein E (VirE) proteins with a C-terminal helicase domain. VirE proteins are found in mobile and transposable elements (MGEs and TEs) associated with bacterial virulence^4^. CAPP_B class proteins are predominantly found in type III CRISPR-Cas operons and are almost exclusively confined within the phylum *Bacteroidetes*.

Although the bioinformatic analyses described above focused on CRISPR-associated Prim-Pols, a diverse set of directly related CAPP_A and CAPP_B homologues are also present in operons lacking CRISPR-Cas genes. These are typically found in MGEs and TEs, although others were detected in bacteriophage genomes (e.g. *Gordonia* phage gene YP_004934804.1). The most prevalent protein domain architecture of the non-CRISPR CAPPs is similar to the third type of CAPP_A, containing a putative helicase domain, although other protein domain fusions also exist (e.g. PriCT).

### CAPPs are DNA-dependent DNA polymerases

The presence of distinctive Prim-Pol genes within CRISPR-Cas operons is intriguing, especially as little is known about which polymerases are specifically involved in these pathways. The operonic association of CAPPs with Cas genes, such as *Cas1* and *Cas2*, implicates them in CRISPR-Cas adaptation. However, in order to define possible roles for CAPPs in CRISPR-Cas adaptation or expression stages, a comprehensive understanding of their biochemical activities was first required. For this study, we selected *MpCAPP* from a deep sea thermophilic bacteria *Marinitoga piezophila* (Mp) (Marpi_0402 / WP_014295918.1)^28^, located upstream of *Cas1*, *Cas2*, and *Argonaute* genes^12^ and *DbCAPP* from a hot spring thermophilic bacteria *Dysgonamonadaceae bacterium* (Db) (C0T31_04825/WP_101486965.1), located between *Cas1* and *Cas2* genes (Fig. 1c). The genes were codon optimised, expressed as C-terminal MBP fusions in *E. coli* and purified to near homogeneity (Supplementary Fig. 2).

**Fig. 2:**
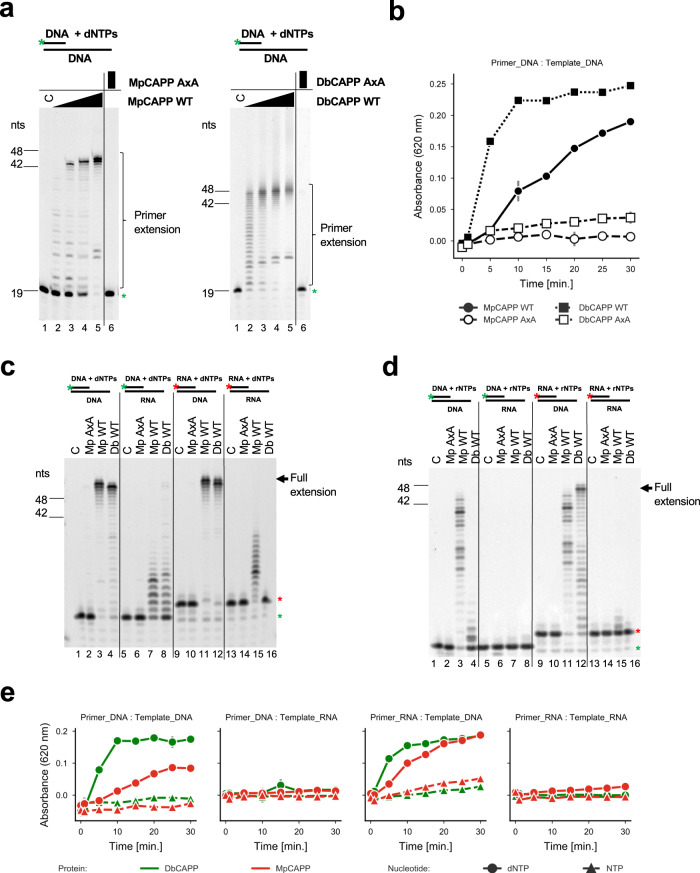
MpCAPP and DbCAPP are DNA-dependent DNA polymerases. **a** MpCAPP possesses DNA polymerase extension activity (left panel). 1, 5, 25 and 125 nM MpCAPP wild-type (WT) or 125 nM D177A + D179A (AxA) mutant protein was added into 30 nM DNA substrate (DNA template + labelled DNA primer) and 100 µM dNTPs in MpPolBuffer. DbCAPP DNA polymerase activity (right panel). 1, 5, 25 and 125 nM DbCAPP wild-type (WT) or 125 nM D86A + D88A mutant (AxA) was added into 30 nM substrate and 100 µM dNTPs in DbPolBuffer. The reactions were incubated at 37 °C for 30 min and resolved by denaturing PAGE. **b** Time course of MpCAPP and DbCAPP polymerase extension activities. In all, 50 nM protein was incubated with 500 nM substrate, 100 µM dNTPs at 37 °C and the generated pyrophosphate was detected using the Malachite Green-based coupled polymerase assay. Circle – MpCAPP WT, open circle – MpCAPP AxA, square – DbCAPP WT, open square – DbCAPP AxA, grey line: standard deviation of *n* = 3 technical replicates. Data are presented as mean values. **c** CAPP polymerase extension activities with dNTPs. Both MpCAPP and DbCAPP have the strongest polymerase activities with DNA templates and dNTPs. In all, 50 nM protein was added into 50 nM substrate and 100 µM dNTPs in MpPolBuffer or DbPolBuffer. The reaction was incubated at 37 °C for 30 min. C – control reaction without protein, Green star – labelled-DNA primer without extension, Red star – labelled-RNA primer without extension, nts – nucleotide length of DNA markers. **d** CAPP polymerase activities with NTPs. MpCAPP and DbCAPP show weaker polymerase activity on DNA template with NTPs. In total, 50 nM protein was added into 50 nM substrate and 100 µM NTPs in buffers as described for panel **c**. The reaction was incubated at 37 °C for 30 min. **e** Time course of MpCAPP and DbCAPP polymerase specificities. 50 nM protein was incubated with 500 nM substrate, 100 µM dNTPs or NTPs at 37 °C and the generated pyrophosphate was detected by using Malachite Green-based coupled polymerase assay. Red – MpCAPP, green – DbCAPP, circle – dNTPs, triangle – NTPs, grey line – standard deviation of *n* = 3 technical replicates. Data are presented as mean values.

To test whether CAPP, like other Prim-Pols, functions as a DNA-dependent DNA polymerase, we performed DNA primer extension assays using fluorescently labelled oligonucleotide primers. Denaturing gel electrophoresis analysis confirmed that both MpCAPP and DbCAPP are active DNA polymerases (Fig. 2a). Both CAPPs are relatively active enzymes, compared to other Prim-Pols, as most of the DNA synthesis products were fully extended to the length of the 45-mer template strand (Fig. 2a – lanes 5, left and right). A mutation of conserved active site metal binding residues (D177A & D179A; AxA mutation) of MpCAPP and (D86A & D88A; AxA mutation) of DbCAPP abolished their activities (Fig. 2a – lanes 6, left and right). A malachite green-based coupled polymerase assay, which measures the release of pyrophosphate (PPi), was used to verify and support the results of the gel-based assays and confirmed that both enzymes are active DNA polymerases and their active site mutant variants are inactive (Fig. 2b).

MpCAPP is active over a wide temperature range with an optima at ~60 °C (Supplementary Fig. 3a), in line with its preferred growth temperature of 65 °C^28^. As the melting temperature of the primer-template used was 50 °C, MpCAPP appears to stabilise the DNA duplex at higher temperatures. We also tested the divalent metal cation dependency on its primer extension activity and observed that MpCAPP synthesis was most efficient with Mg^2+^ (Supplementary Fig. 3b – lane 4), less with Mn^2+^ or Ni^2+^ (lane 5 and 8) and there was little or no detectable activity with Co^2+^,Ca^2+^, Zn^2+^ or Fe^2+^ (lanes 6, 7, 9 and 10). Combining Mg^2+^ with other divalent metal cations did not significantly increase further MpCAPP’s polymerase activity (Supplementary Fig. 3b – lanes 11–16). DbCAPP’s DNA-polymerase activity was highest in the presence of Mn^2+^ (Supplementary Fig. 3c – lane 5) and slightly lower with Mg^2+^ or Co^2+^ (lane 4 and 9). Other tested divalent metal cations were less stimulating of DbCAPP’s polymerase activity (lanes 6, 7, 8 and 10). Similar to MpCAPP, combining Mg^2+^ with other divalent metal cations did not significantly increase the polymerase activity of DbCAPP (Supplementary Fig. 3c – lane 11–16).

Because of the significant role that RNA plays in CRISPR-Cas pathways, particularly in type III systems, we next tested CAPP’s synthesis activities on RNA substrates. When four different combinations of DNA and RNA were used for primer and template strands of the polymerase substrate in presence of dNTPs, a clear preference of both CAPPs to utilise DNA as a template for incorporating dNTPs was observed (Fig. 2c – lanes 3, 4, 11 and 12). The polymerase activities of MpCAPP and DbCAPP on RNA templates in presence of dNTPs was much lower in comparison to DNA templates (lanes 7, 8, 15 and 16). When all four template – primer combinations were tested in presence of NTPs, MpCAPP and DbCAPP exhibited RNA primer extension activity on DNA templates (Fig. 2d – lane 3, 4, 11 and 12), although significantly lower when compared to dNTPs (Fig. 2c) and very weak or no extension was observed on RNA templates (Fig. 2d – lanes 7, 8, 15 and 16). The catalytic MpCAPP mutant (AxA) exhibited no detectable activity in any of these combinations (Fig. 2c, d – lanes 2, 6, 10 and 14). The malachite green-based polymerase assay was also used to verify CAPPs substrate preferences by measuring their enzymatic activities as a time course for every combination of substrates and CAPPs (Fig. 2e). Both enzymes effectively incorporated dNTPs in presence of DNA template and DNA or RNA primer, the dNTP incorporation was not efficient in presence of RNA template. The incorporation of NTPs was not effective with any substrate combination. In conclusion, both CAPPs exhibited efficient polymerase activity with preference for DNA templates and dNTPs over RNA templates and NTPs.

**Fig. 3:**
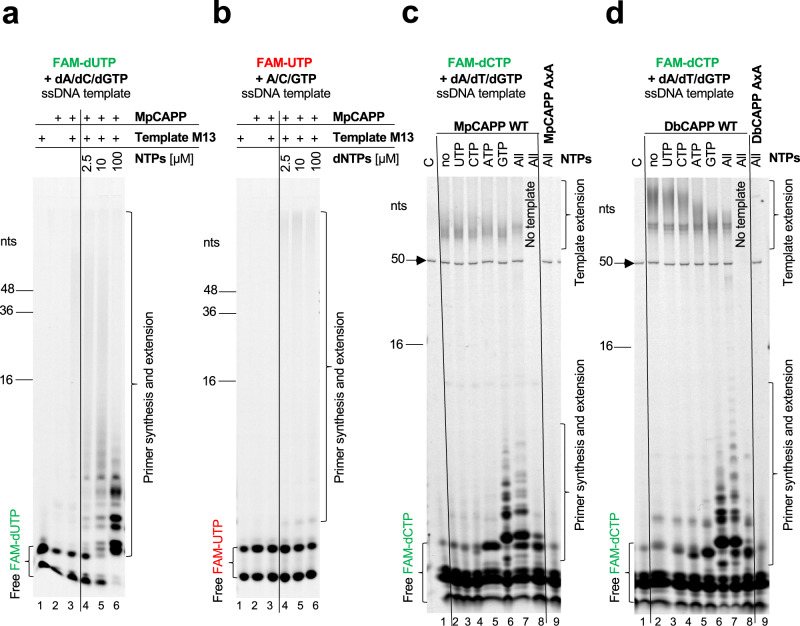
CAPPs require ribonucleotides for primer synthesis. **a** MpCAPP needs both NTPs and dNTPs for primer synthesis. In all, 4 µM MpCAPP wild-type protein was added into 10 ng/µl circular M13 ssDNA substrate in presence of 2.5 µM dNTPs (FAM-labelled dUTP) + 2.5–100 µM non-labelled NTPs and MpPrimBuffer. The reaction was incubated at 50 °C for 30 min and the products were resolved by denaturing PAGE. **b** MpCAPP FAM-labelled UTP incorporation is very poor. In total, 4 µM MpCAPP was added into 10 ng/µl circular M13 ssDNA substrate in presence of 2.5 µM NTPs (FAM-labelled UTP) + 2.5–100 µM non-labelled dNTPs and MpPrimBuffer. The reaction was incubated at 50 °C for 30 min. **c** MpCAPP priming is purine ribonucleotide dependent. In all, 1 µM MpCAPP was added into 1 µM mixed-sequence ssDNA substrate (oKZ53) in presence of 2.5 µM dNTPs (FAM-labelled dCTP) and 1 mM unlabelled NTPs as indicated in the figure. The reaction was incubated at 50 °C for 10 min. C – control reaction without protein, no – no NTPs, all – all NTPs, black arrow – signal of Cy5-labelled template. **d** DbCAPP primase activity is stimulated by addition of purine ribonucleotides. In total, 1 µM DpCAPP protein was added into 1 µM ssDNA (oKZ53) in presence of 2.5 µM dNTPs (FAM-labelled dCTP) and 1 mM unlabelled NTPs in DbPrimBuffer. The reaction was incubated at 50 °C for 10 min. Annotations identical as for panel **c**. nts – nucleotide length of DNA markers. Results shown are representative of three independent repeats (3a–d).

### CAPPs are DNA-dependent DNA primases

To establish whether MpCAPP also possesses DNA-dependent DNA primase activity, the protein was incubated either with circular single-stranded DNA (ssDNA) from M13 phage or short ssDNA oligonucleotides and a mixture of fluorescently labelled and unlabelled dNTPs^29^. However, the enzyme lacked any apparent DNA priming activity, under a wide range of experimental conditions (e.g. different buffers, metal ions, temperatures). A number of known primases initiate primer synthesis with a specific ribonucleotide, which is then extended with dNTPs^30^. We therefore repeated the priming assays in the presence of both, dNTPs (with FAM-dUTP) and increasing concentration of NTPs, using ssDNA M13 as a template. MpCAPP showed very weak primase activity in the presence of only dNTPs (Fig. 3a – lane 3). However, we observed robust primase activity in reactions with both types of nucleotide (NTP and dNTPs) and primase activity was significantly elevated with increasing NTP concentration (lanes 4–6). We noted that products are shorter with increasing concentration of NTPs. As the concentration of dNTPs is relatively low (2.5 µM), most dNTPs are used for priming with increasing concentration of ribonucleotides, therefore we concluded that MpCAPP cannot continue with primer extension.

**Fig. 4:**
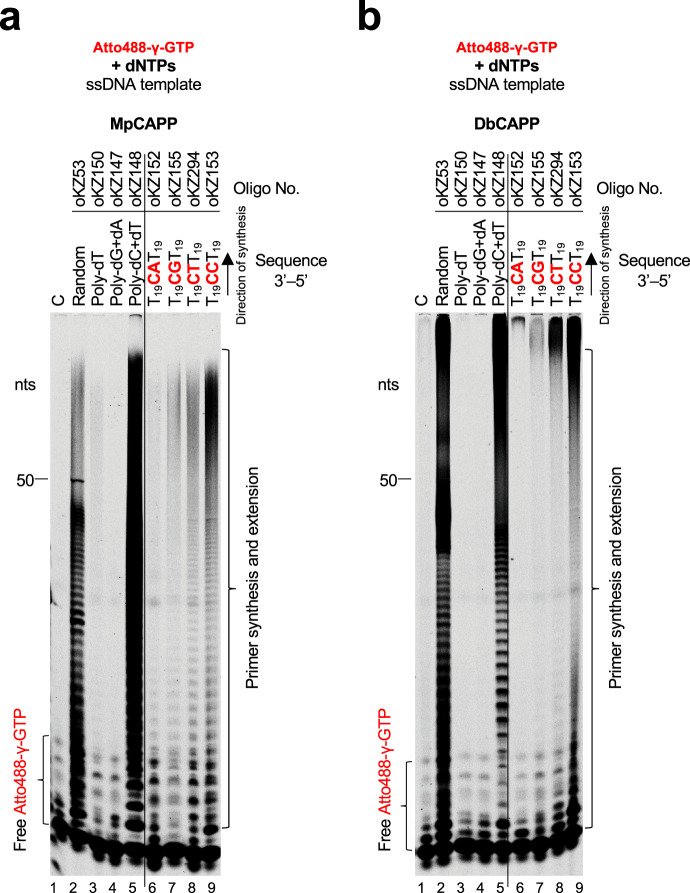
CAPPs initiate primer synthesis with a 5′ ribonucleotide and prefer purines. **a** γ-phosphate labelled GTP incorporation during MpCAPP de novo primer synthesis is sequence-dependent. In total, 1 µM of MpCAPP was added into the reaction with MpPrimBuffer containing 1 µM ssDNA substrates as indicated, 100 µM dNTP mix and 10 µM γ-phosphate Atto488-labelled GTP. The reactions were incubated at 50° for 30 min. The products were resolved on 20% urea-PAGE gel. C – control reaction without protein, nts – nucleotide length of DNA markers. **b** γ-phosphate labelled GTP incorporation during DbCAPP de novo primer synthesis is sequence-dependent. In total, 1 µM of DbCAPP was added into the reaction with DbPrimBuffer containing 1 µM DNA substrates as indicated, 100 µM dNTP mix and 10 µM γ-phosphate Atto488-labelled GTP. The reactions were incubated at 50° for 30 min. The products were resolved on 20% urea-PAGE gel. Annotations identical as for panel **a**. Results shown are representative of three independent repeats (4a, b).

Next, we tested NTPs incorporation during primer synthesis with M13 circular ssDNA and a mix of NTPs and FAM-UTP (2.5 µM) with increasing concentration of unlabelled dNTPs. MpCAPP showed no detectable priming activity in presence of only NTPs (Fig. 3b – lane 3) and this activity increased only slightly with increasing dNTPs concentration (Fig. 3b – lanes 4–6). The results above suggest that CAPP can incorporate ribonucleotides during the first dinucleotide step of primer synthesis and preferentially uses dNTPs for the following extensions, in agreement with CAPP’s polymerase activity (Fig. 2c–e). A ribonucleotide may be incorporated as the first base of the primer as described for protein ORF904 from archaeal plasmid pRN1^30^. To determine if MpCAPP preferentially incorporates a specific NTP, we repeated the assays with a single type of ribonucleotide added with the dNTP pool using random sequence single-stranded oligonucleotide templates (Fig. 3c) or circular ssDNA M13 (Supplementary Fig. 4a). We observed that the presence of GTP, and to a lesser extent ATP, supported robust priming activity on both ssDNA templates (Fig. 3c – lanes 5 and 6 and Supplementary Fig. 4a – lanes 3 and 6). However, only weak stimulation of primer synthesis was observed in the presence of CTP or UTP (Fig. 3c – lane 3 and 4, Supplementary Fig. 4a – lanes 4 and 5), in comparison with reactions containing dNTPs only (Fig. 3c – lane 2, Supplementary Fig. 4a – lane 2). Primer synthesis in presence of GTP was at a comparable level to that observed in the presence of all NTPs, which indicates GTP is the major stimulator of the primase activity (Fig. 3c – compare lanes 6 and 7). The catalytic mutant (AxA) of MpCAPP showed no detectable primase activity (lane 9). DbCAPP’s DNA-dependent DNA primer synthesis activity on oligonucleotide ssDNA was also stimulated in presence of GTP (Fig. 3d, lane 6), and to lesser extent by ATP (lane 5) and weak stimulation was observed in presence of UTP or CTP (lanes 3 and 4), indicating a similar ribonucleotide preference to MpCAPP. Although DbCAPP priming on M13 ssDNA (Supplementary Fig. 4b) appeared to be potentially less ribonucleotide specific. In conclusion, these results establish that CAPP priming activity is stimulated by the presence of purine ribonucleotides, with preference for GTP over ATP.

The optimal temperature for MpCAPP’s primase activity was ~ 50 °C (Supplementary Fig. 5). MpCAPP exhibited elevated DNA primase activity with Co^2+^ ions and slightly lower with Mn^2+^ (Supplementary Fig. 6a – lane 5 and 9). Zn^2+^ and Mg^2+^ supported very weak primase stimulation on their own (lanes 4 and 7) and Ca^2+^, Ni^2+^ and Fe^2+^ did not support primase activity (lanes 6, 8 and 10). Notably, the addition of a small amount of Zn^2+^ (100 µM), in presence of Mg^2+^ (10 mM), induced the strongest stimulation of MpCAPP primase activity out of all conditions tested (lane 13). Increasing concentrations of Zn^2+^, up to a limit of 100 µM in the presence of 10 mM Mg^2+^ and DNA template, stimulated its priming activity (Supplementary Fig. 7 – lanes 7–10). Increasing the concentration of GTP also had a similar stimulatory effect on priming (Supplementary Fig. 7 – lanes 3–5). Interestingly, MpCAPP catalysed dinucleotide formation even in the absence of a ssDNA template, albeit significantly lower than in the presence of ssDNA (Supplementary Fig. 6b). The strongest dinucleotide formation without template was observed in presence of Zn^2+^ (Supplementary Fig. 6b – lane 7), less so with Co^2+^ and also with a mixture of 10 mM Mg^2+^ and 100 µM Zn^2+^ (lanes 9 and 13) (Note, Fe^2+^ cations exhibited degradation products likely the result of a Fenton reaction). The markedly strong effect of Zn^2+^ on MpCAPP’s primase activity suggests that this divalent ion is somehow enhancing the initial step of priming, e.g. dinucleotide formation potentially due to the inhibition / slowing down of the CAPP polymerase activity (Supplementary Fig. 3b – lane 13).

Next, we investigated how the type of nucleic acid polymer affected MpCAPP primer synthesis activity in presence of GTP (100 µM) and observed that it was much higher with DNA substrates and dNTPs (Supplementary Fig. 8 – lane 2). MpCAPP created NTP-dNTP dinucleotides with and without RNA templates (lanes 5 and 13) at a similar level, suggesting that MpCAPP cannot efficiently prime on RNA templates. Moreover, it was unable to prime using just NTPs, suggesting that at least one dNTP is necessary for the first dinucleotide synthesis, contrary to replicative primases that can only utilise NTPs during this process. However, once an RNA primer is present, MpCAPP can efficiently extend it using dNTPs. The catalytic mutant (AxA) was again inactive on all tested substrates (Supplementary Fig. 8 – lanes 3, 6, 9 and 12). Together, these findings establish that CAPPs are DNA-dependent DNA primases that preferentially initiate primer synthesis using GTP.

### CAPPs incorporate a ribonucleotide as the first base in primer synthesis

MpCAPP and DbCAPPs show both DNA-dependent DNA polymerase and DNA-dependent primase activities (Figs. 2c–e and 3, and Supplementary Fig. 8). However, they require a ribonucleotide with preference to GTP for efficient priming (Fig. 3c, d). To determine whether a ribonucleotide is incorporated by MpCAPP and DbCAPPs as the first or second base during the first dinucleotide synthesis, we used γ-phosphate Atto488-labelled GTP (Atto488-γGTP) for priming reactions with a mixed-sequence short ssDNA template and dNTPs (Fig. 4a, b, lane 2). During synthesis, the γ-phosphate of the incoming nucleotide is cleaved off during the release of PPi produced during phosphodiester bond formation. Therefore, the strongly visible oligomers produced by MpCAPP and DbCAPP in lane 2 (Fig. 4a, b, respectively) must consist of primers with GTP incorporated as the first base of the newly synthesised primers. These results unequivocally establish that dinucleotide synthesis begins with ribonucleotide incorporation as the first 5′ nucleobase.

To determine, if the first incorporation step in de novo primer synthesis is sequence-dependent, we repeated the priming assay on different homopolymeric and heteropolymeric substrates. Abundant primer synthesis was observed with templates containing deoxycytidines (Fig. 4a, b, lanes 5–9) and little activity observed with templates lacking deoxycytidines (Fig. 4a, b, lanes 3–4). This indicates that GTP is the first base incorporated during primer synthesis and this incorporation is dependent on base-pairing.

De novo primer synthesis begins with the formation of a dinucleotide, which is subsequently extended to form a primer^5^. Primases often initiate dinucleotide formation from a recognition sequence composed of preferred di/tetra nucleotides^31^. We used a library of ssDNA oligonucleotide templates, to define any preferred sequences for CAPP priming, containing different base combinations placed in the center of poly-deoxythymidine flanking sequences. As GTP is the preferred first base incorporated during dinucleotide formation (Fig. 3c, d), we used a library containing all sequence variants of 3′-T_18_X**C**XXT_18_-5′ and tested which sequence from this library stimulates MpCAPP and DbCAPP priming. The most relevant results, (shown in Fig. 4a, b and Supplementary Fig. 9a, b), indicate that the preferred template sequence for initiation of MpCAPP priming is 3′-X**C**CT/C-5′, and for DbCAPP it is 3′-X**C**C/T-5′ (initiating deoxycytidine is shown in bold).

Next, we tested if MpCAPP requires riboguanosine α-, β- or γ-phosphates for the initiation of primer synthesis. The most favourable priming was observed in the presence of GTP (Supplementary Fig. 10, lane 3), only slightly reduced with GDP and even more decreased with GMP (lanes 4 and 5, respectively). No priming was observed with guanosine (lane 6). These results suggest that at least one (α-) phosphate is essential for the stabilisation of the guanosine nucleotide in the active site of MpCAPP for primer initiation.

### CAPPs possess synthesis-dependent strand displacement activity

**Fig. 5:**
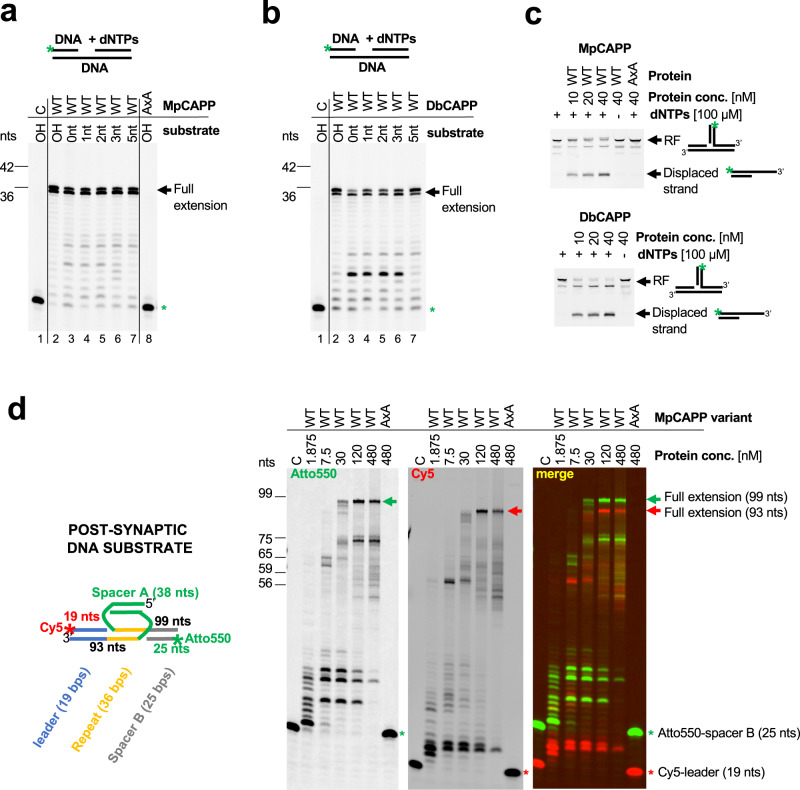
CAPPs possess synthesis-dependent strand displacement activity. **a** MpCAPP possesses strong strand displacement activity. Polymerase assay was performed with 50 nM protein and 50 nM dsDNA substrates, containing a gap of the indicated lengths (0 – nick, 1, 2, 3, 5 or OH – over-hang). C – no enzyme, WT – wild-type, AxA – active site mutant, Green star – labelled-DNA primer without extension. **b** DbCAPP possesses strong strand displacement activity. Polymerase assay was performed on dsDNA substrates as described in panel **a**. Annotations identical as for panel **a**. **c** MpCAPP is able to dismantle a half-site integration intermediate – replication fork using its strand-displacement activity. Assay was performed with the indicated concentrations of proteins with or without dNTPs in presence of replication fork (RF) substrate. The products were resolved on native PAGE gel. WT (wild type) and AxA (active site mutant). **d** Left panel – Schematic representation of post-synaptic DNA substrate used in the MpCAPP-displacement assay (right). Right panels – MpCAPP is able to fully displace Cas1-Cas2 post-synaptic DNA substrate. Displacement assay was performed on 30 nM post-synaptic substrate at 50 °C for 30 min. C – no enzyme, WT – wild-type, AxA – active site mutant, Green signal – Atto550, Red signal – Cy5, Red star – Cy5-labelled leader, Green star – Atto550-labelled Spacer B, Red and green arrows – full extension products after CAPP strand displacement synthesis (Cy5- and Atto550-labelled, respectively), nts – nucleotide length of DNA markers. Results shown are representative of three independent repeats (**a**–**d**).

Having characterised the basic biochemical activities of MpCAPP and DbCAPP, we next addressed the role(s) CAPPs may play in CRISPR-Cas related processes. Due to *CAPPs* proximity to *Cas1* and *Cas2* genes, we hypothesised three possible roles it may undertake in the adaptation process: (1) MpCAPP’s primase and polymerase activities may be involved in prespacer synthesis; (2) it may separate the two strands of the fully integrated CRISPR repeat, inserted by Cas1-Cas2, creating ssDNA gaps; and, finally, (3) it may fill in these gaps. In order to separate the two strands of a CRISPR repeat (2), following full integration of a spacer by the Cas1-Cas2 complex, MpCAPP would require robust strand displacement activity. To investigate this possibility, we performed primer extension assays on dsDNA substrates containing a nick or gaps of different lengths. We observed that both MpCAPP and DbCAPP are able to efficiently extend, in all cases, the primers to the end of the template strand (Fig. 5a, b). This establishes that these CAPPs have strong strand displacement activity, which only requires a 3′ hydroxylated nick in the DNA strand to initiate the synthesis. Next, to investigate if these CAPPs were also able to operate on substrates resembling Cas1-catalysed DNA spacer integration intermediates, we examined their ability to disassemble a half-site integration construct. Notably, both CAPPs were able to efficiently perform displacement synthesis, dismantling this substrate and thus producing a dsDNA product (Fig. 5c). The observations that dNTPs were needed during the process, and AxA mutant had no detectable activity, confirmed the involvement of CAPP’s DNA polymerase activities in strand displacement. Finally, we tested if MpCAPP was able to displace Cas1-Cas2 post-synaptic DNA substrate containing full-length repeat and spacer sequence (Fig. 5d – left). Indeed, MpCAPP extended the 3′ ends of the Cy5-labelled leader and Cy3-labelled spacer B up to the ends of spacer A, fully displacing the post-synaptic substrate (Fig. 5d – right). The structural validity of this integration intermediate was confirmed using a ssDNA specific nuclease, which indicated that the substrate is annealed as shown (Supplementary Fig. 11). CAPP’s robust strand-displacement activity may also help to explain the observed synthesis of DNA primers longer than the original template in the primase assay (see Figs. 3c, d and 4). After the primer is freed by a second round of synthesis on the same template, it could be further extended after annealing to a different site.

### MpCAPP forms homodimers

**Fig. 6:**
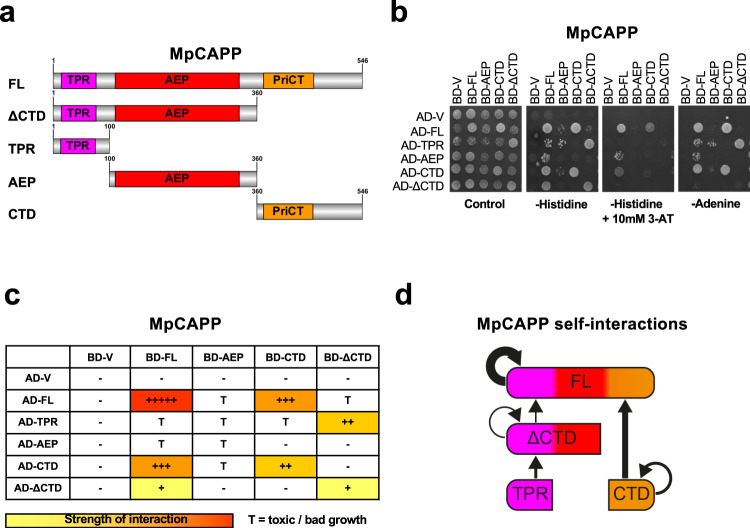
MpCAPP domains involved in self-association. **a** Schematic representaion of MpCAPP with its domains highlighted – three tetratricopeptide repeat (TPR) repeats in magenta, archaeo-eukaryotic primase (AEP) catalytic core (aa. ~110–340) in red and primase C-terminal (PriCT), containing conserved cysteine residues, in orange found in C-terminal domain (CTD). **b** MpCAPP shows self-association in the yeast two-hybrid assay. Full-length (FL) MpCAPP and its fragments (ΔCTD – aa. 1-360, TPR – aa. 1-100, AEP – aa. 100-360, and CTD – aa. 360-546) were either fused with GAL4 DNA-binding domain (BD) or its activation domain (AD) and their interaction with the indicated counterparts or empty vector (V) was established on selective plates lacking leucine, tryptophan and histidine or adenine. Addition of 10 mM 3-amino-triazole (3-AT) was also used to increase stringency of the histidine reporter. Results shown are representative of three independent repeats. **c** Schematic summarising the interactions observed: +++++ stands for very strong interaction, +++ for strong interaction, ++ for medium interaction, + for weak interaction and – for no interaction. T stands for toxic growth. **d** Graphical summary of the observed interactions.

MpCAPP is composed of three discernible domains (Figs. 1c and 6a). An N-terminal TPR domain, a Prim-Pol catalytic domain (AEP) and a PriCT-like domain found in the C- terminal domain (CTD). As TPR domains often promote homodimer or oligomer formation, we tested the possible self-association of MpCAPP using the yeast two-hybrid assay (Y2H). We prepared constructs where the full-length gene or various fragments (TPR: 1-100, ΔCTD: 1-360, AEP: 100-360, CTD: 360-546) (Fig. 6a) were either fused to the yeast transcription factor GAL4’s DNA-binding domain (BD) or its activation domain (AD). The various combinations of AD and BD protein fusions were expressed together in yeast and potential interactions between MpCAPP’s domains were assayed. This is facilitated by bringing together of the two GAL4 domains, leading to HIS3 and ADE2 expression and resulting in growth on selective plates either without histidine (plus 3-amino-1,2,4-triazole (3-AT) – a competitive inhibitor of the HIS3 gene product) or adenine. This assay revealed interactions between the MpCAPP fragments (Fig. 6b). We detected strong interactions between full-length proteins, slightly less interaction between FL protein and CTD. There was moderate interaction between CTDs and similar interaction was observed between AD-TPR and BD-ΔCTD. Finally, there was observed weak interaction between ΔCTDs and between BD-FL and AD-ΔCTD (summarised in Fig. 6c, d). The MpCAPP-TPR-BD fusion was self-activating and was therefore left out of the analysis, while the rest of the fusions did not show interactions with the GAL4 AD or BD domains on their own (AD-V or BD-V, respectively). Some combinations of co-transformed constructs caused toxicity in yeast. Although the results of these problematic combinations were reproducible, we excluded them from our summary (Fig. 6c – toxicity = T) as these results could be false positives. The observation that the interaction between FL MpCAPPs was reproducibly the strongest, and that the interactions between CTDs and between TPR and ΔCTD showed moderate interaction, suggests that CAPP self-association has at least two interaction surfaces. One interaction surface is located at CTD allowing interaction between two CTDs and the second surface is at TPR interacting with ΔCTD (Fig. 6d).

All our biochemical assays were performed using MpCAPP with a C-terminal MBP fusion, as MpCAPP is insoluble without this fusion. Therefore, we tested if the MBP fusion is influencing MpCAPP self-association in Y2H. The AD-MpCAPP fused to MBP (AD-MpCAPP-MBP) interacted with BD-MpCAPP (without MBP), similarly to AD-MpCAPP (without MBP) suggesting little or no effect of the MBP fusion on the MpCAPP self-association (Supplementary Fig. 12a). MpCAPP-MBP fusion has a size of ~108 kDa but it eluted from a size-exclusion chromatography column (Superdex 200 10/300) at a predicted mass of ~300–400 kDa protein (Supplementary Fig. 12b), suggesting that MpCAPP-MBP forms multimeric complexes.

### MpCas1-Cas2 mediates CRISPR array specific spacer integration

**Fig. 7:**
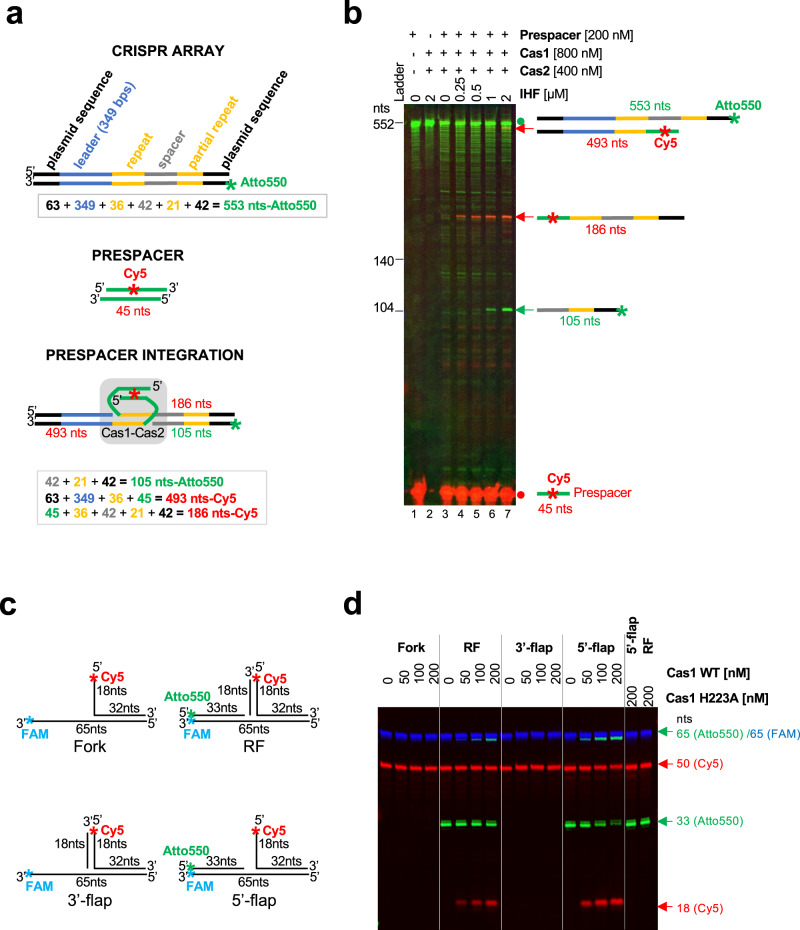
MpCas1 site-specific integration and disintegration activities. **a** Schematic representation of the MpCRISPR array, before and after prespacer integration, used in the Cas1-integration assay (panel **b**). **b** 26 nM CRISPR array (PCR-synthetised) was incubated with wild-type Cas1 (WT) in presence of Cas2, increasing concentration of IHF and 200 nM prespacer (Cy5-labelled) in integration buffer (10 mM Bis-Tris Propane; pH 7, 10 mM MgCl_2_, 100 mM NaCl, 0.5 mM TCEP and 0.1 mg/ml BSA) for 90 min at 50 °C. After Proteinase K digestion, the products were resolved on denaturing urea-PAGE. Green signal – Atto550 (CRISPR array), Red signal – Cy5 (prespacer) Red dot – prespacer, Green dot – CRISPR array without any integration, Red and green arrows– products after prespacer integration. **c** Schematic representation of branched substrates used in Cas1 transesterification assay (panel d). RF – replication fork. **d** MpCas1 prefers transesterification of the 5′-flap over RF and other tested branched structures. Increasing concentration of Cas1 was incubated with 100 nM branched substrates in buffer containing 10 mM Bis-Tris Propane; pH 7, 10 mM MgCl_2_, 10 mM NaCl and 0.3 mg/ml BSA for 30 min at 50 °C. After Proteinase K digestion the products were resolved by denaturing urea-PAGE. Green signal – Atto550; Red signal – Cy5; Blue signal – FAM, nts – nucleotide length of DNA markers. Results shown are representative of three independent repeats (7b, d).

Having identified that *CAPPs* are genetically associated with *Cas1* and *Cas2* genes in a wide range of species (Fig. 1b), we next tested the functionality of this CRISPR integrase complex. As a representative CAPP-associated Cas1-Cas2 complex, we chose to test *Marinitoga piezophila* Cas1 (MpCas1) (Marpi_0403/ WP_014295919.1) and Cas2 (MpCas2) (Marpi_0404/ WP_014295920.1) for their ability to integrate a prespacer into a partial MpCRISPR arrays in fluorescently labelled PCR fragment depicted in (Fig. 7a and Supplementary Note 1). These proteins were purified to near homogeneity (Supplementary Fig. 2). At first, the MpCas1-Cas2 complex was inefficient in the correct prespacer integration into the first repeat next to the leader sequence (105 nts – Atto550, 186 and 493 nts – Cy5) (Fig. 7b, lane 3 and Supplementary Fig. 13). Integration host factor (IHF) was previously identified as an important factor that increases specific Cas1-Cas2 integration^32–34^ by bending the leader sequence of the CRISPR array. IHF is a heterodimer composed of IHFα and IHFβ subunits in *E. coli*, although BLAST searches identified only a single *E. coli IHF* sequence homologue in the *M. piezophila* genome (Marpi_0943 / WP_014296428). Nevertheless, we confirmed that *M. piezophila* IHF protein (MpIHF) homodimerized using Y2H (Supplementary Fig. 14). We expressed and purified MpIHF (Supplementary Fig. 2) and used it, together with MpCas1-Cas2, in integration assays (Fig. 7b, lanes 4–7 and Supplementary Fig. 13a, b). The correct specific integration was significantly improved by the presence of MpIHF and the yield of this integration product was enhanced with increasing MpIHF concentration. Notable, these results do not allow us to distinguish between full prepacer integration and half-site integration. In the case of full integration, one prespacer would be integrated into top and bottom strand of one dsDNA molecule. If it was half-site integration, two prespacers weould be integrated into both strands on two different dsDNA molecules independently of each other.

Plasmid-based integration assays, followed by PCR, confirmed that MpIHF is important for correct (half-site) prespacer integration into the CRISPR array. We discovered, that similar to *Sulfolobus solfataricus* (*S. solfataricus*) Cas1 and Cas2 (SsCas1 and SsCas2)^35^, MpCas2 was not essential for the prespacer integration (Supplementary Fig. 15).

Next, we tested how leader length influenced prespacer integration in vitro. In the *E. coli* type I-E CRISPR system, only 60 bp of the leader proximal to the first repeat is required for adaptation^36^. In contrast, *S. solfataricus* Cas1-Cas2 required a 500-bp leader^35^. *M. piezophila* type IIIB CRISPR has three different CRISPR arrays in close proximity of the MpCAPP-Cas1-Cas2-Ago operon (Fig. 1a). Approximately 400 bps in close proximity of the first repeat are highly conserved (Supplementary Note 2 and 3). We tested three different truncations of the leader sequence of the first CRISPR1 array, located upstream of MpCas1 and MpCas2 genes (next to MpCAPP) (Fig. 1c, CAPP-TPR operon), in our integration assay. A: full-length leader sequence– 498 bps, B: 298 bps and C: 132 bps (Supplementary Fig. 16 and 17 and Supplementary Note 1). There was no effect of the examined leader length on the top strand leader-repeat junction integration. However, we observed significant and reproducible negative effects of leader truncations on the prespacer integration into the bottom strand at the repeat-spacer junction (Supplementary Figs. 16 and 17). The most efficient prespacer integration into the bottom strand occurred only with CRISPR array containing full-length leader (CRISPR array A).

Recently, a two-step model for Cas1-Cas2 prespacer integration was proposed^37,38^. The first nucleophilic attack (the first integration step) occurs on the top strand at the leader-repeat junction and the second nucleophilic attack (the second integration step) is made on the bottom strand at the repeat-spacer junction. Based on this model, we conclude that an *M. piezophila* CRISPR leader sequence, longer than 298 bps, is required only for the second prespacer integration step. Here, we demonstrate in vitro a prespacer integration mechanism of a Type III CRISPR system that possesses a canonical Cas1-Cas2 complex.

### MpCas1 exhibits disintegration activity

*E. coli* and *S. solfataricus* Cas1 have disintegration activities on branched DNA substrates, which is Cas2 independent^38,39^, preferentially on replication forks or 5′-flaps^38^. During disintegration, the 5′-flap (or replication fork) undergoes a transesterification reaction that cleaves the single-stranded 5′-flap at the junction and concomitantly ligates the resulting nicked DNA strand. We investigated if MpCas1 possesses disintegration activity using four different fluorescently labelled branched DNA substrates: simple fork, Replication fork (RF), 3′-flap and 5′-flap (Fig. 7c, d). Indeed, MpCas1 showed some transesterification activity on replication fork and significantly stronger activity on a 5′-flap structure, in agreement with activities observed in a previous study^38^. Mutation of MpCas1’s conserved active site histidine (H223) to alanine (H223A) abolished MpCas1’s transesterification activity (Fig. 7d).

### CAPPs interact with Cas1-Cas2

**Fig. 8:**
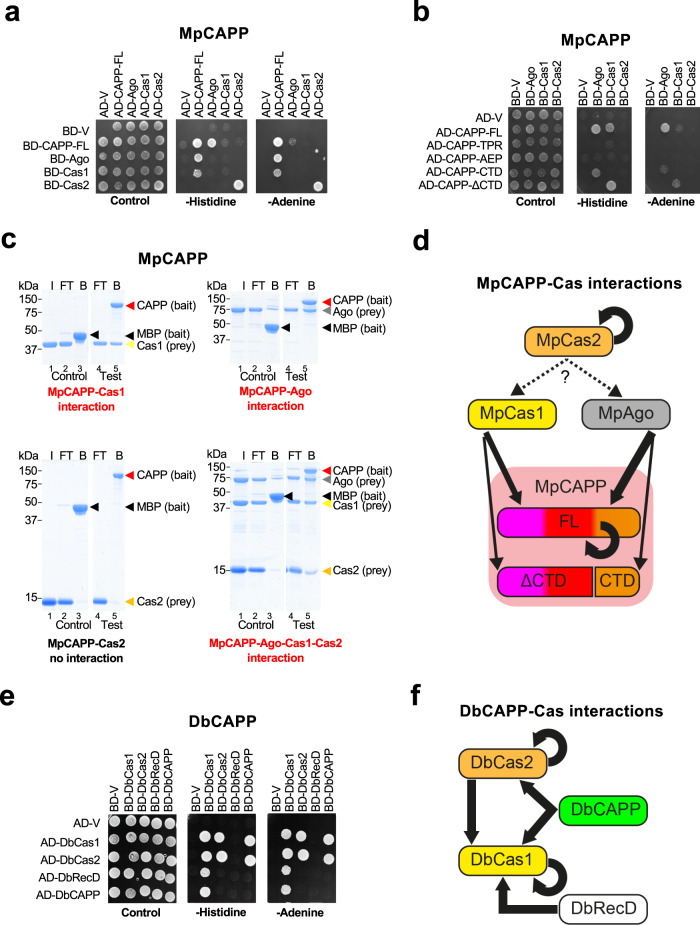
CAPPs form a complex with Cas1 and Cas2. **a** MpCAPP interacts with MpAgo and MpCas1 in yeast two-hybrid assays (Y2H) and MpCas2 self-associates. GAL4 DNA-binding domain (BD), activation domain (AD), empty vector (V). **b** Interactions between MpCAPP full-length (FL) and itself or fragments (FL – aa. 1-546, TPR – aa. 1–100, AEP – aa. 101–359, CTD – aa. 360–546, ΔCTD – aa. 1–359), MpCas1, MpCas2 and MpAgo were evaluated by Y2H. **c** MpCAPP forms a complex with MpCas1, MpCas2 and MpAgo proteins and directly interacts with MpAgo and MpCas1 in pull-down assays. Bait (MpCAPP-MBP or MBP) was pre-bound to the amylose beads and washed before adding prey (MpAgo, MpCas1 and/or MpCas2). I – prey input (the same input was used for the test and control experiment), FT – flow-through, B – bound protein. Test and control samples were run on the same gel (Supplementary Fig. 19). **d** Graphical summary of MpCAPP-Cas1-Cas2-Ago interactions. **e** DbCAPP interacts with DbCas1 and DbCas2, DbCas1 interacts with DbCas2 and DbRecD in Y2H. DbCas1 and DbCas2 self-associate. **f** Graphical summary of DbCAPP-Cas1-Cas2-RecD interactions. TPR – tetratricopeptide repeat – magenta domain, AEP – archaeo-eukaryotic primase – red domain, PriCT – primase C terminal – orange domain, MBP – maltose binding protein. Results shown are representative of three independent repeats in 8a–c, e.

Although our findings so far suggest possible roles for CAPPs in CRISPR-Cas mechanisms, they do not in themselves establish that these replciases function in this pathway. Therefore, evidence of any direct interplay between CAPPs and other CRISPR-Cas proteins is required to further support this hypothesis. Due to their conserved operonic proximity, we considered MpCas1, MpCas2 and MpArgonaute (MpAgo) as prime candidates to interact and function with MpCAPP. Y2H assays were used to test interactions of MpCAPP with its immediate operonic neighbours (Cas1, Cas2 and Ago). We observed that MpCAPP strongly interacts with MpAgo, less so with MpCas1, and MpCas2 strongly self-associates (Fig. 8a). Notable, MpCAPP AD-fusions interacted with MpAgo and MpCas1 stronger than BD-fusions, as these protein fusions can often influence interactions differently. Further fragment analysis of MpCAPP’s interactions revealed that MpCas1 interacts with MpCAPP’s ΔCTD fragment (aa. 1–360) and MpAgo interacts with MpCAPP CTD (aa. 360–546) (Fig. 8b).

To validate these observed MpCAPP interactions, we performed pull-down analysis using recombinant proteins in vitro. MpCAPP-MBP fusion interacted directly with MpCas1 and MpAgo, but not with MpCas2 (Fig. 8c). However, when using MpCAPP-MBP bait with a mixture of MpCas1, MpCas2 and MpAgo preys, all proteins were detected in the bound fraction (Fig. 8c), suggesting indirect interactions of MpCas2 with the MpCAPP, either via MpCas1 or MpAgo. Together, these findings establish that MpCAPP forms a bespoke complex with its operonic partners (Ago, Cas1 and Cas2) (Fig. 8d).

DbCAPP abilities to self-associate and interact with its close operonic partners (DbCas1, DbCas2 and DbRecD) was also analysed using Y2H. DbCAPP is composed of an AEP catalytic domain (VirE-N) (~ aa. 1–220) and a diverse smaller C-terminus (~ aa. 220–337) and it was found not to self-associate (Fig. 8e). DbCas1 and DbCas2 do self-associate and also interact with each other, forming a canonical Cas1-Cas2 complex. DbCas1 interacts with both DbCAPP and another proximal operonic gene product, a putative RecD-like helicase (Fig. 1c, CAPP_B operon; Fig. 8e, f). The DbCAPP-DbCas1 interaction was also confirmed using direct pull-down assays (Supplementary Fig. 18). In summary, both MpCAPP and DbCAPP form specific complexes with their respective Cas1 and Cas2 neighbouring genes (Fig. 8d, f) suggesting that CAPP-Cas1-Cas2 complex formation may be a common feature for type III CRISPR-Cas systems that contain CAPPs.

## Discussion

**Fig. 9:**
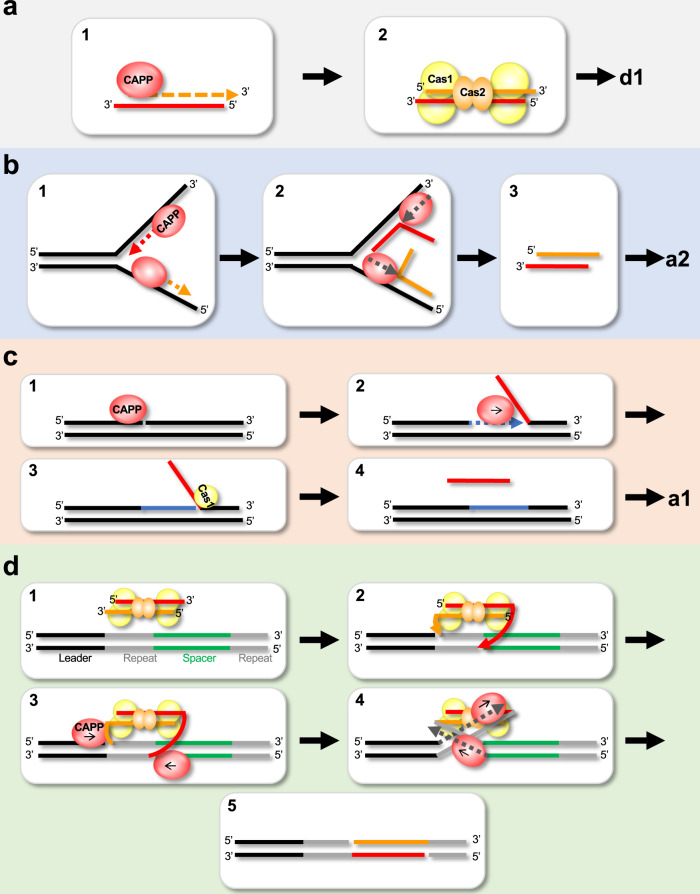
Prospective roles of CAPP during CRISPR-cas adaptation. **a–c** Roles CAPPs may play during prespacer synthesis. **a** First role of CAPP during prespacer synthesis: 1. CAPP starts de novo primer synthesis and extension on the short ssDNA (e.g. nuclease degradation, CAPP synthesis or Cas1-transesterification ssDNA products). 2. Cas1-Cas2 complex binds the CAPP-synthesised dsDNA (prespacer) ready for integration. **b** Second CAPP role in prespacer synthesis: 1. Two CAPP molecules start synthesis on both DNA strands. 2. Strand displacement synthesis by another CAPP displaces the newly synthesised strands. 3. These complementary ssDNA strands could be annealed by Cas1-Cas2 complex (and the process could continue as described in panel a2). **c** Third role of CAPP in prespacer synthesis: 1. CAPP recognises nicked DNA. 2. CAPPs displacement synthesis activity creates 5′-flap structures. 3. Cas1 recognises 5′-flap and creates short ssDNA substrate via its transesterification activity and the process continues as described in panel a1. **d** Role of CAPP in the resolution of the post-synaptic complex. 1. Prespacer bound by Cas1-Cas2 complex and docks with the CRISPR array in readiness for new spacer integration. 2. Cas1 cleaves the first repeat strand (next to the leader) by transesterification and ligates the 3′-prime strands of the prespacer. 3. CAPP binds to the nicks on the Cas1-Cas2 spacer integration intermediate. 4. CAPP starts strand displacement synthesis of the repeat strands, resolving the post-synaptic complex. 5. The new spacer is ligated to complete spacer integration into the CRISPR array.

Many of the major activities associated with CRISPR-Cas mechanisms have been characterised, including nucleolysis and integration, but a number of key enzymes involved in these pathways remain elusive. Although genetic studies have implicated host polymerases in some CRISPR-Cas systems^40–42^, CRISPR-specific replicases have not been described so far. Here, we report the characterisation of a family of CRISPR-Cas associated Prim-Pols called CAPPs, which possess a remarkable variety of synthesis activities. *CAPPs* operonic association with *Cas1* and *Cas2* genes, together with the functional data presented here, firmly supports the hypothesis that these replicases likely undertake key roles in the adaptation process. CAPPs are unusual replicases possessing both primase and polymerase activities, preferentially synthesising DNA products and exhibiting strong synthesis-dependent strand-displacement activity. Given these characteristics and its CRISPR-Cas operonic location, we propose that CAPP’s synthesis activities may be directly involved in prespacer synthesis during the adaptation phases of this adaptive immunity process (Fig. 9).

The first possible scenario (a) is dependent on the activities of RecBCD/addAB. It was shown that functional RecBCD (Gram-negative)^15^ or addAB complexes (Gram-positive)^43^ are important for new spacer integration into CRISPR arrays. However, as the degradation products of RecBCD/addAB nucleolysis results in short ssDNA and the nuclease activity of RecBCD is dispensable for spacer acquisition^17^, it is still unclear how dsDNA prespacers arise. Given CAPPs can initiate primer synthesis on short ssDNAs and fully extend complementary strands, they are strong candidates for producing dsDNA prespacers for subsequent Cas1-Cas2 integration using ssDNA degradation products of RecBCD or other nucleases as templates (Fig. 9a).

The second scenario (b) is independent of RecBCD/addAB. CAPPs may have evolved by acquiring a viral replicative Prim-Pol (e.g *Gordonia* Phage CAPP-like proteins) thus inheriting an enzyme that can readily access viral replication origins and also act on replication forks of phage that do not possess their own primases^44^. At the viral replication fork, two CAPP molecules could potentially act like a standard viral primase and start synthesis on both DNA strands, but in opposite directions. In the case of MpCAPP, this synthesis may be stimulated by self-dimerisation, as proposed for the herpes-simplex virus 1 (HSV1) helicase primase^45^. Once synthesis finishes (probably due to the lack of helicase activity), strand displacement would allow another CAPP molecule to displace the newly synthesised strand produced by the other CAPP (Fig. 9b). Cas1-Cas2 complex strand annealing activity could then be used to pair the complementary products thus creating a dsDNA prespacer^19^. Alternatively, one displaced primer can act as a template for second round of CAPP priming/extension synthesis to produce the second complementary DNA strand. In both scenarios, the resulting dsDNA prespacer would then be handed over to Cas1-Cas2 for processing and integration.

The third possible scenario (c) is Ago/Can2-dependent. It was shown that MpAgo acts as an RNA-guided ssDNA nuclease^12^ and Can2 (VC1899 gene in Fig. 1a – *T. islandicus*, *C. gasimii* and *D. bacterium*) is a putative dsDNA nickase^46^. Ago/Can2 may assist, not only by cleaving the invading virus nucleic acids, but also stimulating prespacer formation by providing CAPP with nicked DNA for displacement synthesis (Fig. 9c). CAPP would then access the nick and displace one strand by strand-displacement synthesis, creating a 5′-flap substrate. The 5′-flap might be cleaved by another protein with nuclease/transesterification activity, e.g. Cas1^38^ (Fig. 7d).

Once CAPP hands over the newly synthesised prespacer to Cas1-Cas2, their association probably persists. We propose that CAPPs may also facilitate the completion of the integration process by disentangling the entwined structure of the fully integrated spacer-repeat intermediate^34,42,47^ using displacement synthesis to both relax and complete replication of this intermediate (Fig. 9d). The remaining nicks would then be ligated to complete this process of spacer acquisition by the CRISPR array. CAPPs displacement synthesis must be blocked at the beginning of the integrated spacer sequence during this process and Cas1-Cas2 binding may assist in this regard^19,42^. There is a consensus that a synthesis activity is requisite to fill in gaps created by the Cas1-Cas2 integration process in all CRISPR systems. We propose that CAPPs may fulfil this important role in some type III systems and also assist in prespacer acquisition/synthesis during CRISPR adaptation. The discovery of CAPPs participation in the acquired immunity provided by some CRISPR-Cas systems further expands the repertoire of enzymes involved in these mechanisms and implicates both synthesis and displacement activities in this adaptive process. How CAPPs and their partners specifically participate in CRISPR-Cas processes, such as adaptation, is currently under investigated. Further studies are also required to establish if other bespoke or host primase/polymerase activities undertake equivalent roles in other CRISPR systems.

## Methods

### Bioinformatic analysis

The sequences of the AEP domain of *Marinitoga sp. 1137* (APT75355) (Msp) or full-length *Dysgonamonadaceae bacterium* (PLB86576) (Db) were used as queries for the distant homologues search. Five rounds of PSI-BLAST^24^ were conducted, using BLAST + 2.10.1 standalone package^48^ (cut-off value *E* < 0.1), on the non-redundant (nr) protein BLAST database downloaded from NCBI^25^ on 2020/08/27. The Msp and Db derived datasets were processed separately. A BASH 5.0^49^ script was used to extract all accession numbers from the BLAST results table to create a list of 20 protein accession numbers around each BLAST hit (40 in total). These protein accession number lists were used to extract protein names for each accession number from the nr protein BLAST database and then each list was queried for the keyword “CRISPR”. All “CRISPR” keyword positive lists of accession numbers were validated by downloading corresponding genomic feature tables from NCBI^25^ using BASH scripts and EDirect utilities^50^. A region of 20 gene neighbours surrounding each BLAST hit (40 in total) was extracted from the feature table and queried for the keyword “Cas1”, creating a subset of regions with both CAPP and CRISPR-Cas1 genes present. All validated CAPP protein sequences from Msp and Db BLAST search were pooled together and a t-coffee^51^ multiple sequence alignment was made and a bootstrapped (*n* = 100) phylogenetic tree was build, using the neighbour joining method and the Kimura protein maximum likelihood method in CLC Main Workbench (Qiagen). All the metadata for the annotation of the phylogenetic tree were extracted using EDirect utilities and BASH scripts from data available at NCBI. For the CRISPR gene frequency heatmap table, all protein names from the validated genomic regions were pooled together per position relevant to CAPP using a BASH script and the occurrence of keywords was computed in Excel. The final table was created in Python 3.9.0^52^ using matplotlib^53^ library.

### Cloning, expression and purification of recombinant proteins

The sequences of all used genes from *Marinitoga piezophila* (except MpCas1) and *Dysgonamonadaceae bacterium* were codon optimised for expression in *E. coli* (see Supplementary Note 4). All cloning details and all used primers are listed in (Supplementary Data 3).

All *M. piezophila* proteins CAPP WT (C-terminal MBP), CAPP AxA (C-terminal MBP), Cas1 WT, Cas1 Mutant (H223A), Cas2 and IHF were expressed from plasmids pKZ43, pKZ60, pPK238, pKZ61, pPK244 and pKZ59, respectively, in the BL21 (DE3) *E. coli* strain. The transformed cell cultures were grown in standard TB medium to OD_600_ of 0.8–1. Expression of all proteins was induced by adding IPTG (Isopropyl β-d-1-thiogalactopyranoside) to a final concentration of 1 mM, followed by 3 h incubation at 37 °C.

MpCAPP purification: cell paste was resuspended in buffer A (50 mM HEPES pH 7.5, 500 mM NaCl, 10% (v/v) glycerol, 1 mM TCEP (Tris(2-carboxyethyl)phosphine) and 10 mM imidazole) containing protease inhibitors. The suspension was sonicated and cleared by ultracentrifugation and the supernatant was mixed with Amintra® Cobalt NTA Affinity Resin (Expedeon) for 60 min at 4 °C. The resins were then extensively washed first in buffer A, followed by buffer A containing 2 M NaCl and finally with buffer A containing 500 mM NaCl again. Proteins were eluted in buffer A containing 300 mM imidazole. Elution fractions were pooled, loaded onto amylose resins (NEB) and incubated for 60 min at 4 °C. Amylose resins were washed in amylose wash buffer (50 mM HEPES pH 7.5, 500 mM NaCl, 10% (v/v) glycerol and 1 mM TCEP) and the protein was eluted with addition of 10 mM maltose. Elution fractions were pooled, concentrated in Vivaspin 20 (Sartorius), aliquoted, frozen in liquid nitrogen and stored at −80 °C.

MpCas1, MpCas2 and MpIHF purification: Cell paste was resuspended in buffer B (50 mM HEPES pH 7.5, 250 mM NaCl, 10% (v/v) glycerol and 0.5 mM TCEP) containing protease inhibitors. Suspension was sonicated and cleared by ultracentrifugation. After addition of imidazole to a final concentration of 10 mM, the supernatant was mixed with Amintra® Cobalt NTA Affinity Resin (Expedeon) for 40 min at 4 °C. The resins were then extensively washed in the same buffer. Proteins were eluted in buffer B containing 300 mM imidazole. Elution fractions were pooled, loaded onto a 5-ml HiTrap Q HP column (Cytiva) and the resulting flow-through was immediately loaded onto a 5-ml HiTrap SP FF column (Cytiva). The SP column was developed with a 50-ml gradient of 250–600 mM NaCl in buffer B. The peak fractions were pooled, concentrated in Vivaspin 20 (Sartorius), aliquoted, frozen in liquid nitrogen and stored at −80 °C.

MpAgo was expressed and purified using a modified protocol from Kaya et al.^12^ pET-6His-MBP-MpAgo (Addgene) was transformed into and expressed in *E. coli* strain BL21(DE3)pLysS. Cells were grown in Turbo Broth medium at 37 °C to an OD_600_ of 1.0, expression was induced via addition of IPTG to a final concentration of 0.4 mM and incubated at 16 °C while shaking for a further 16 h. Cell pellets were resuspended in 50 mM HEPES pH 7.5, 250 mM NaCl, 10 mM imidazole, 0.5 mM TCEP, 0.05% Triton X-100, 0.2 mM PMSF (phenylmethylsulfonyl fluoride) and Turbo DNase (Invitrogen). Sonication was used to lyse the cells, and clarified lysate were bound to 5 mL Amintra CoHis resin (Expedeon). The resins were washed (50 mM HEPES pH 7.5, 250 mM NaCl, 10 mM imidazole and 0.5 mM TCEP), and bound protein was eluted in wash buffer containing 300 mM imidazole. The His-MBP affinity tag was removed with HRV 3C protease, diluted into 50 mM HEPES pH 7.5, 150 mM NaCl and 5% glycerol, applied onto a 5 mL Heparin HiTrap column (GE Life Sciences), and eluted via a linear NaCl gradient of 150–1200 mM. The protein sample was further purified via size-exclusion chromatography, using a HiLoad 16/60 Superdex 200 column (GE Life Sciences) in 20 mM HEPES pH 7.5, 300 mM NaCl, 5% glycerol and 0.5 mM TCEP. Fractions containing purified protein were concentrated, flash-frozen in liquid nitrogen and stored at a −80 °C freezer.

DbCAPP protein (WT and AxA mutant) was found most stable with C-terminal MBP fusion in a customised pET28(+)-a vector named pETSTTHMH. The activity of MBP-DbCAPP fusion was comparable with N-Terminal His tag purification alone (not shown). Proteins were expressed in BL21(DE3) in TB media supplemented with trace metals^54^, induced with 1 mM IPTG at 37 °C for 3 h.

DbCAPP purification: cell paste was resuspended in 50 mM HEPES pH 7.5, 0.5 M NaCl, 10% glycerol, 30 mM imidazole, 0.5 mM TCEP, sonicated and cleared by ultracentrifugation. The supernatant was loaded on HisTrap column (Cytiva) and eluted in the same buffer with gradient to 0.5 M imidazole. The major peak was collected and loaded onto Streptactin XT HC (IBA) and eluted with the same buffer with 50 mM biotin added. The peak fractions were concentrated in Vivaspin 20 (Sartorius) and resuspended to 50% glycerol, aliquoted, frozen in liquid nitrogen and stored at −80 °C.

### Polymerase assay

In the polymerase assays, 20 μl reactions contained, 30 or 50 nM substrate (indicated in the figure legends) with: FAM-labelled primers in combinations: DNA primer – DNA template (Oligo oPK405 + oPK404), DNA primer – RNA template (oPK405 + oPK406), RNA primer – DNA template (oPK407 + oPK404) or RNA primer – RNA template (oPK407 + oPK406) (all oligonucleotides are listed in Supplementary Data 3), and 100 μM dNTPs. Reactions were supplemented with MpCAPP or DbCAPP (concentrations indicated in the figure legends) in their respective MpPolBuffer (10 mM Bis-Tris Propane; pH 7, 10 mM MgCl_2_, 10 mM NaCl_2_ and 0.5 mM TCEP) or DbPolBuffer (10 mM Bis-Tris; pH 6.5,10 mM NaCl, and 1 mg/ml BSA) and incubated at 37 °C for 30 min. Reactions were quenched with 20 μl stop buffer (60% formamide, 6 M urea, 5 mM EDTA, 0.025% SDS) and boiled for 3 min before electrophoresis on a denaturing gel containing 15% polyacrylamide (AA: bis-AA; 19 :1, 7 M urea and 1× TBE buffer). The gel was run at 25 W for 90 min in 1× TBE. Extended fluorescent primers were imaged using a Typhoon FLA 5100 scanner (Cytiva).

### Malachite Green-based coupled polymerase assay

A Malachite Green polymerase assay was designed as a coupled enzyme assay^55^. CAPPs release pyrophosphate (PPi) upon phosphodiester bond formation and a thermostable inorganic pyrophosphatase, TiPP (M0296L, NEB) converts PPi to inorganic phosphate (Pi), which is then detected by the Malachite Green Phosphate Assay Kit (MAK307, Merck). A master-mix containing following components: 10 mM Tris-HCl; pH 7, 1 mM MgCl_2_, 100 µM dNTP mix or NTP mix, 500 nM substrate, 50 nM CAPP and 0.1 mU/µl TiPP was dispensed into 190 µl samples and incubated at 37 °C with shaking for indicated time. After the incubation samples were quenched on ice with 20 mM EDTA, for every timepoint, the samples were split into three technical replicates (60 µl) in a clear 96-well plate (Nunc) pre-filled with 20 µl ddH_2_O and 20 µl of Malachite Green reagent was added and mixed well. After 10 min incubation at a room temperature, absorbance (620 nm) was recorded using ClarioStar (BMG Labtech). Background values of reactions of respective nucleotide mix without CAPP were subtracted and means and standard deviations were calculated in Excel and plotted with Matplotlib and Seaborn. Substrates used were non-fluorescent versions of substrates used in the polymerase assay. DNA primer – DNA template (Oligo oPK405noFAM + oPK404), DNA primer – RNA template (oPK405noFAM + oPK406), RNA primer – DNA template (oPK407noFAM + oPK404) or RNA primer – RNA template (oPK407noFAM + oPK406) (all oligonucleotides are listed in Supplementary Data 3).

### Primase assay

MpCAPP primase assay: 20 μl reaction contained MpPrimBuffer (10 mM Bis-Tris Propane; pH 7, 0.5 mM TCEP, 10 mM MgCl_2_ and 100 µM ZnCl_2_), either 10 ng/µl circular M13 ssDNA (NEB) or 1 µM ssDNA substrate, MpCAPP proteins, unlabelled NTPs and dNTPs (NEB) and either FAM-labelled dUTP, FAM-labelled UTP, FAM-labelled dCTP or γ-phosphate Atto488-labelled GTP (NU-803-6FM, NU-821-6FM, NU-809-5FM, NU-834-488, Jena Bioscience) at combinations and concentrations indicated in the figure legends. All reactions were incubated at 50 °C for 30 min (unless indicated otherwise). The reactions were then cleaned either using the Oligo Clean & Concentrator kit (Zymo Research) or ethanol precipitation (added 20 µl of stop solution: 0.6 M KOAc, ph 5.5, 40 mM EDTA and mixed with 100 µl 100% ethanol, pelleted DNA at 17,000 rcf, 4 °C for 10 min, aspirated and resuspended in 20 µl of loading buffer: 96% formamide, 0.3% Ficoll 400, 10 mM EDTA). The samples were boiled for 3 min before electrophoresis on a denaturing gel containing 20% polyacrylamide (AA: bis-AA; 19 :1, 8 M urea and 1× TBE buffer). The gel was run at 25 W for 115 min in 1× TBE. The fluorescent primase products were imaged using a Typhoon FLA 5100 scanner (Cytiva).

DbCAPP primase assay: 20 μl reaction contained DbPrimBuffer (10 mM Bis-Tris; pH 6.5, 10 mM MnCl_2_, 0.5 mM TCEP), either 50 ng circular M13 ssDNA (NEB) or 1 µM ssDNA substrate, with DbCAPP proteins and nucleotides (same as for MpCAPP) at combinations and concentrations indicated in the figure legends. The reaction was incubated at 50 °C for 30 min (unless indicated otherwise) followed by 95 °C for 3 min. The samples were cleaned by ethanol precipitation and loaded on a denaturing gel containing 20% polyacrylamide (AA: bis-AA; 19 :1, 8 M urea and 1× TBE buffer). The gel was run at 25 W for 115 min in 1× TBE. The fluorescent primase products were imaged using a Typhoon FLA 5100 scanner (Cytiva).

### DNA strand-displacement assay

Strand displacement assays involving gapped DNA substrates were performed in 20 µl volume. Substrates were formed by annealing oligonucleotide oNB1 and oNB2 together with one oligonucleotide from oNB3–7 (Supplementary Data 3)^56^. In total, 50 nM substrate was mixed together with 100 µM dNTPs, 50 nM MpCAPP or DbCAPP in MpPolBuffer or DbPolBuffer, respectively. The reactions were incubated at 37 °C for 30 min and stopped by stop buffer. The products were boiled for 3 min, resolved on 15% urea-PAGE in 1× TBE for 90 min at 25 W and imaged using a Typhoon FLA 5100 scanner (Cytiva).

The strand displacement of a half-site integration construct was performed in 10 μl reactions containing 50 mM Tris-HCl pH 7.5, 1 mM MgCl_2_, 1 mM DTT, 0.1 mg/ml BSA, 40 nM fluorescein-labelled integration template (Supplementary Data 3 – oligonucleotides oKZ349–352) in the presence or absence of 100 μM dNTPs. Proteins at concentrations indicated in the figure legends were added and reactions were incubated at 37 °C for 30 min. The reaction was stopped by addition of 1 µl of 1% SDS and 0.8 U of proteinase K (NEB) and incubated at 37 °C for 30 min. After adding of 3 µl of 24% Ficoll 400 to the samples, the reaction products were resolved by electrophoresis on a 10% native polyacrylamide gel in 1 × TBE buffer. Gel was scanned using Typhoon FLA 5100 imager (Cytiva).

Displacement assays with post-synaptic substrates was performed in presence of 30 nM substrate (oligonucleotides oKZ366–369 – Supplementary Data 3), with proteins at concentrations indicated in figure legends added, 200 µM dNTPs in 10 mM Bis-Tris Propane; pH 7, 10 mM MgCl_2_, 10 mM NaCl, 0.5 mM TCEP and 1 mg/ml BSA. The reactions were incubated at 50 °C for 30 min. The reaction was stopped by adding of 1 µl of 1% SDS, 0.8 U of Proteinase K (NEB) and 0.5 µl of 0.5 M EDTA; pH 8 followed by incubation at 50 °C for 30 min and 12 µl of stop buffer was added into the reaction. The samples were boiled for 3 min and resolved on 10% urea-PAGE in 1× TBE for 90 min at 25 W and imaged using a Typhoon FLA 5100 scanner (Cytiva).

### Yeast two-hybrid assay

Full-length MpCAPP and its fragments (amino acids 1-100, 1-360, 100-546, 100-360, 360-546), MpCas1, MpCas2, MpAgo, DbCAPP, DbCas1, DbCas2 and DbRecD genes were cloned into vectors containing GAL4 activation domain (pGADT7 or pAD-Gate2^57^) or vectors containing Gal 4 DNA-binding domain (pGBKT7 or pBD-Gate2^57^) For details see Supplementary Data 3. These constructs were co-transformed into the *Saccharomyces cerevisiae* strain PJ69-4A and plated on selective medium lacking leucine and tryptophan. Individual colonies were inoculated in YPAD medium and incubated over night at 30 °C. This culture was diluted 10-times and drop tests performed on media lacking leucine, tryptophan, histidine or adenine. 3-amino-1,2,4-triazole (3-AT) was also added to the medium lacking leucine, tryptophan and histidine to increase the stringency of the drop test. Plates were scanned after 3 days of incubation at 30 °C.

### In vitro MpCas1-Cas2 prespacer integration assay on DNA fragments

A CRISPR array from *M. piezophila* (Supplementary Note 1 (top CRISPR array)) was synthesised by PCR (Q5 High Fidelity DNA polymerase, NEB): template – pKZ73, primers – oKZ354 and oKZ342. The PCR product was resolved on 2% agarose gel and gel-extracted before use in the assays. The principle of the in vitro MpCas1-Cas2 prespacer integration assay was described previously^42^. MpCas1 and MpCas2 were preincubated with prespacer (annealed oKZ347 and oKZ106) for 5 min at room temperature in buffer containing 10 mM Bis-Tris Propane; pH 7, 10 mM MgCl_2_, 100 mM NaCl, 0.1 mg/ml BSA and 0.5 mM TCEP. At the same time, MpIHF was preincubated with the CRISPR array substrate for 5 min at room temperature. Cas1-Cas2-prespacer complex and MpIHF with CRISPR array complex were mixed (10 µl total volume) and further incubated for 60 min at 50 °C. The reaction was stopped by adding of 1 µl of 1% SDS, 0.8 U of Proteinase K (NEB) and 0.5 µl of 0.5 M EDTA and further incubated 50 °C for 5 min. In total, 13 µl of stop buffer (60% formamide, 6 M Urea, 5 mM EDTA, 0.025% SDS) was added into the samples. The samples were boiled for 3 min and loaded on 8% urea-PAGE (AA: bis-AA; 19 :1, 7 M urea and 1× TBE buffer). The gel was run at 25 W for 90 min in 1× TBE and imaged using a Typhoon FLA 5100 scanner (Cytiva).

### MpCas1 transesterification assay

The principle of this assay was described previously^38^. In all, 20 µl reaction containing increasing concentration of MpCas1 with 100 nM branched substrates (Fork: oKZ53 + oKZ54; Replication fork (RF): oKZ53 + oKZ54 + oKZ56 + oKZ58; 3′-flap: oKZ53 + oKZ54 + oKZ58 or 5′-flap: oKZ53 + oKZ54 + oKZ56) in transesterification buffer (10 mM Bis-Tris Propane; pH 7, 10 mM MgCl_2_, 10 mM NaCl and 0.3 mg/ml BSA) was incubated for 30 min at 50 °C. The reaction was stopped by adding of 0.8 U of Proteinase K (NEB) and 1 µl of 0.5 M EDTA followed by incubation at 37 °C for 30 min. In all, 20 µl of Stop buffer (60% formamide, 6 M Urea, 5 mM EDTA, 0.025% SDS) was added into each reaction before 3 min boiling. The products were resolved on 15% denaturing Urea-PAGE (AA: bis-AA; 19 :1, 7 M urea and 1× TBE buffer). The gel was run at 25 W for 2 hours in 1× TBE. Extended fluorescent primers were imaged using a Typhoon FLA 5100 scanner (Cytiva).

### In vitro pull-down assay

The assay was performed in pull-down buffer containing 25 mM HEPES; pH 7.5, 100 mM NaCl, 50 mM KCl, 3 mM MgCl_2_, 5% glycerol, 0.5 mM TCEP and 0.1% IGEPAL CA-630. In all, 20 µg of bait MpCAPP (C-terminal MBP) or MBP (expressed from oPINM vector – Addgene^58^) in 200 µl of pull-down buffer was preincubated with 50 µl of amylose resins (NEB) at 4 °C for 30 min. The resins were washed three-times with 500 µl of pull-down buffer and 20 µg of prey proteins MpCas1, MpCas2 and/or MpAgo were added to the resins in 200 µl volume of pull-down buffer. The resins were incubated at 4 °C for 45 min and washed three-times with 500 µl of pull-down buffer. The proteins were eluted from the resins with 40 µl of 4-times SDS Sample loading buffer. In total, 5 µl of each fraction was loaded on 12% SDS-PAGE which was Coomassie stained after the run.

### DNA and RNA oligonucleotide templates

Sequences and modifications of the used synthetic oligonucleotides are shown in Supplementary Data 3. Double-stranded templates and different branched structures were prepared by mixing equimolar amounts of the corresponding oligonucleotides in buffer containing 50 mM Tris pH 7.5, 100 mM NaCl, 10 mM MgCl_2_, heating at 95 °C for 3 min and cooling slowly to room temperature. The annealed products were then run on native PAGE (1× TBE), cut from the gel, extracted by overnight diffusion, ethanol precipitated and the pellet was dissolved in water.

### Reporting summary

Further information on research design is available in the Nature Research Reporting Summary linked to this article.

## Supplementary information

Supplementary Information

Supplementary Data 1

Supplemenatary Data 2

Supplementary Data 3

Description of Additional Supplementary Files

Reporting Summary

## Data Availability

All relevant data are included with the paper, as Supplementary Information, Supplementary Data are available upon request from the authors. Uncropped blots and gel images are available as Supplementary Fig. 19.
