# Supplementary material for: CRISPR-Associated Primase-Polymerases are implicated in prokaryotic CRISPR-Cas adaptation
Source: Nat Commun. 2021 Jun 17;12:3690. doi: 10.1038/s41467-021-23535-9 (PMC8211822; doi:10.1038/s41467-021-23535-9)
Supplement: Supplementary file 1 — Supplementary Information [file 41467_2021_23535_MOESM1_ESM.pdf]

Supplementary information

CRISPR-associated Primase-Polymerases are implicated in prokaryotic CRISPR-Cas adaptation

Zabrady et al.

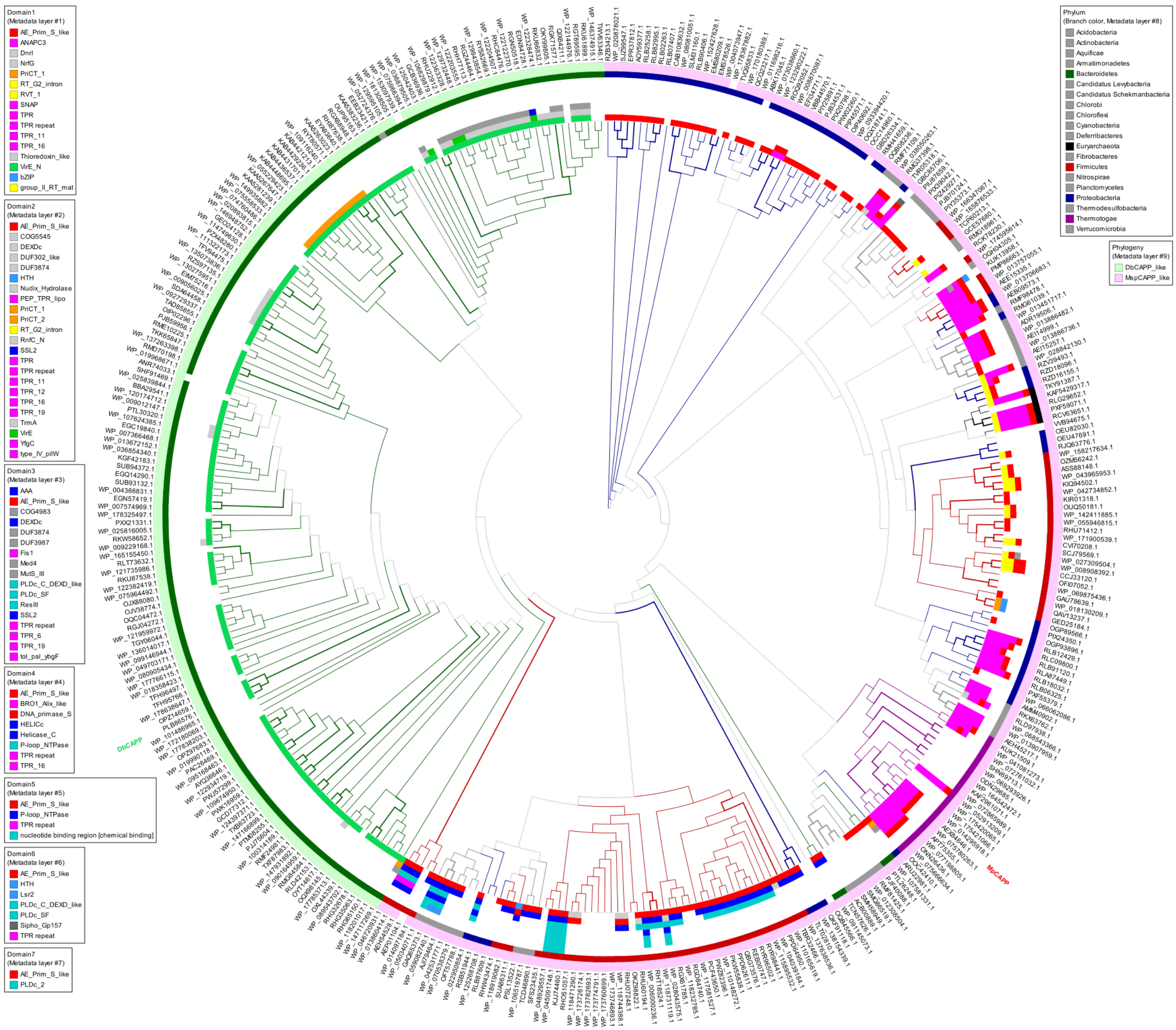

Supplementary Figure 1: A full phylogenetic tree from multiple sequence alignment of all CAPP proteins found. Branch colours and strong outer ring colours indicate phyla from NCBI taxonomy database. Inner ring is composed of strong coloured protein domain annotations of CAPP proteins from NCBI CDD database. Protein domain annotations are ordered in metadata layers consecutively, as found in the database. Light outer ring colours indicate BLAST hits datasets, Db (Supplementary Data 1) and Msp (Supplementary Data 2). NCBI protein accession numbers indicate leaf nodes. . Bootstrap values of 100% are indicated with bold branches.

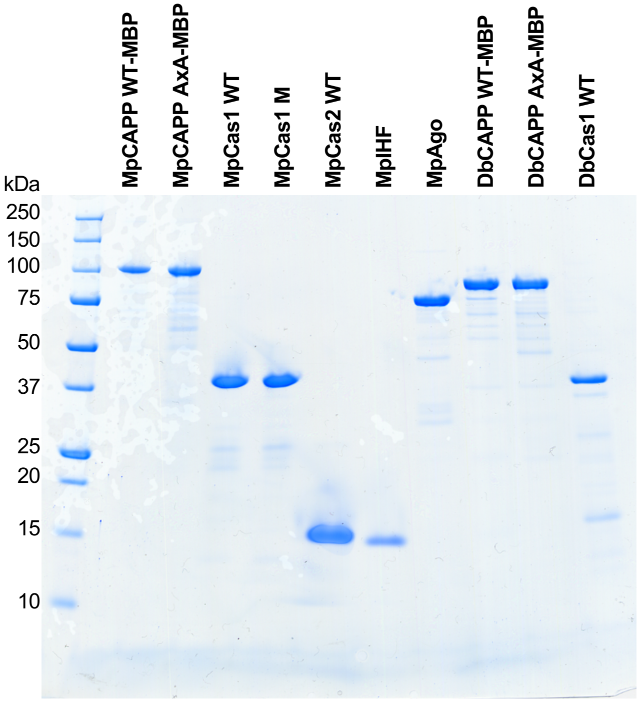

Supplementary Figure 2: SDS-PAGE analysis of purified proteins used in this study. 2 μg of purified proteins. were resolved on 4–12 % Bis-Tris SDS-PAGE in MES buffer and stained with Coomassie Blue. Results shown representative of two independent repeats.

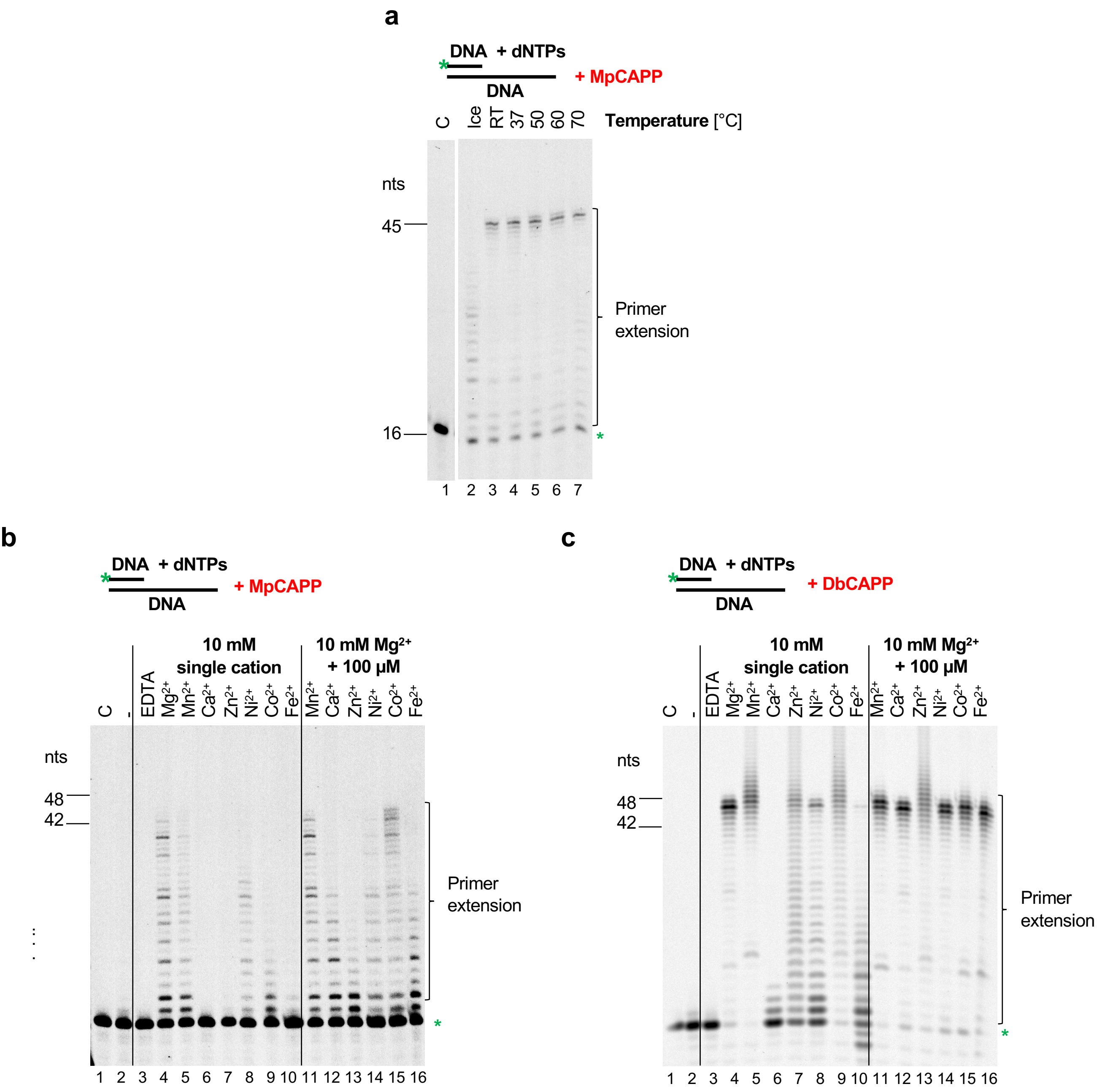

Supplementary Figure 3

Supplementary Figure 3: Effect of divalent metal cations and temperature on CAPP's polymerase activities. (a) MpCAPP optimal temperature for polymerase activity is 50–70 °C. 25 nM MpCAPP was added into 30 nM substrate (DNA template + FAM-labelled DNA primer) and 100 μM dNTPs in MpPolBuffer. The reaction was incubated at temperature as described in the Figure for 30 min. C: reaction without enzyme, Green star: labelled-DNA primer without extension. **(b)** MpCAPP shows the highest polymerase activity in presence of Mg^{2+}. 10 nM MpCAPP was added into 30 nM substrate (DNA template + FAM-labelled DNA primer) and 100 μM dNTPs in 10 mM Bis-Tris Propane; pH7, 10 mM $NaCl_2$ and 0.5 mM TCEP with depicted concentrations of $MgCl_2$, $MnCl_2$, $CaCl_2$, $NiCl_2$, $ZnCl_2$, $CoCl_2$, and $FeCl_2$. The reaction was incubated at 37 °C for 30 min. **(c)** DbCAPP shows the highest polymerase activity in presence of Mn^{2+}. 50 nM DbCAPP was added into 50 nM substrate (DNA template + FAM-labelled DNA primer) and 100 μM dNTPs in buffer containing 10 mM Bis-Tris; pH 6.5,10 mM NaCl, and 1 mg/ml BSA and indicated concentration of metal ions. The reaction was incubated at 37 °C for 30 min. -: no added ions or EDTA, C: reaction without enzyme, Green star: FAM-labelled DNA primer without extension. Oligonucleotide (nts) length markers are shown on the left of the gel. Results shown representative of three independent repeats (3a–c).

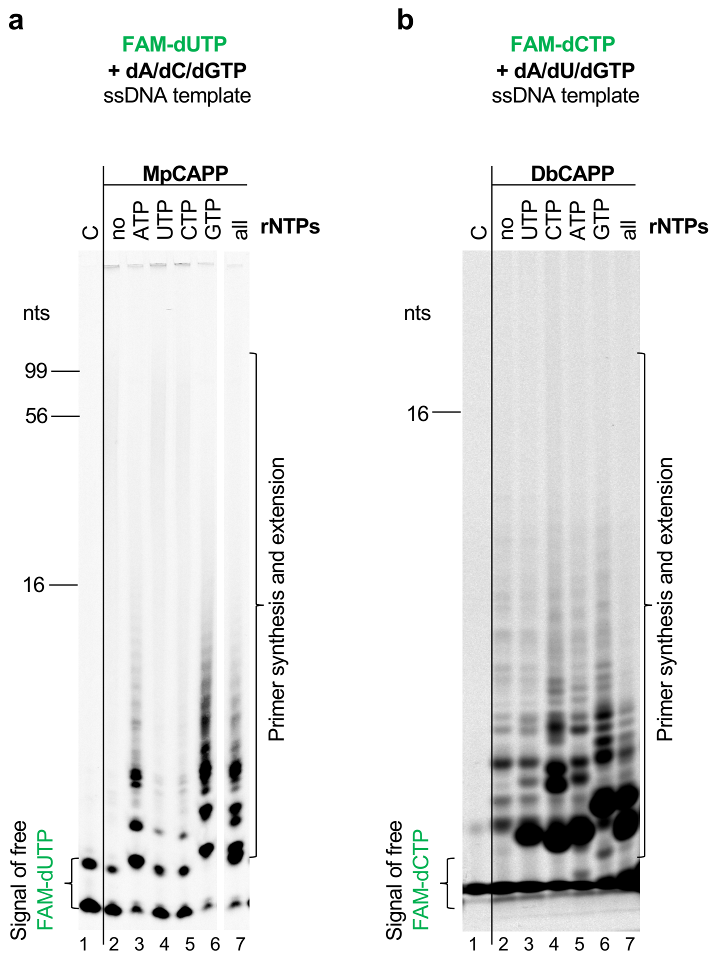

Supplementary Figure 4: CAPP needs purine ribonucleotides for the primer synthesis. (a) The MpCAPP priming is purine ribonucleotide dependent. 4 μM MpCAPP was added into 10 ng/μl circular M13 ssDNA substrate in presence of 2.5 μM dNTPs (dUTP labelled) and 100 μM individual non-labeled rNTPs (lane 3-6) or rNTP mix (lane7) and MpPrimBuffer. The reaction was incubated at 50 °C for 30 min. **(b)** DbCAPP primase activity is stimulated in presence of all ribonucleotides. 1 μM DpCAPP protein was added into 50 ng circular M13 ssDNA in presence of 10 μM dNTPs (FAM-labelled dCTP) and 1 mM rNTPs (10 mM Bis-Tris; pH 6.5, 10 mM $MnCl_2$). The reaction was incubated at 50°C for 10 min. C: control reaction without protein, no: reaction without ribonucleotide. Oligonucleotide (nts) length markers are shown on the left of the gel. Results shown representative of three independent repeats (4a,b).

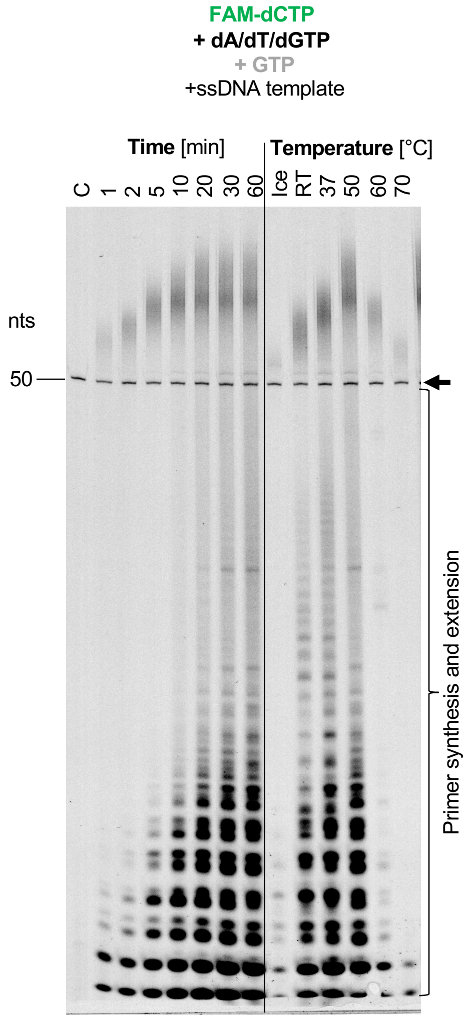

Supplementary Figure 5: Optimal priming conditions for MpCAPP. MpCAPP is most efficient in temperature around 50 °C. 1 μM MpCAPP was added into 1 μM Cy5-labelled oligonucleotide template (oKZ53) in presence of 2.5 μM dNTPs (dCTP was FAM-labelled), 100 μM GTP and MpPrimBuffer. The reaction was incubated ether at 50 °C for indicated time (1–60 min) (left) or at different temperature for 30 min. (right). C: control reaction without protein. Black arrow: signal of Cy5-labelled template. Results shown representative of three independent repeats.

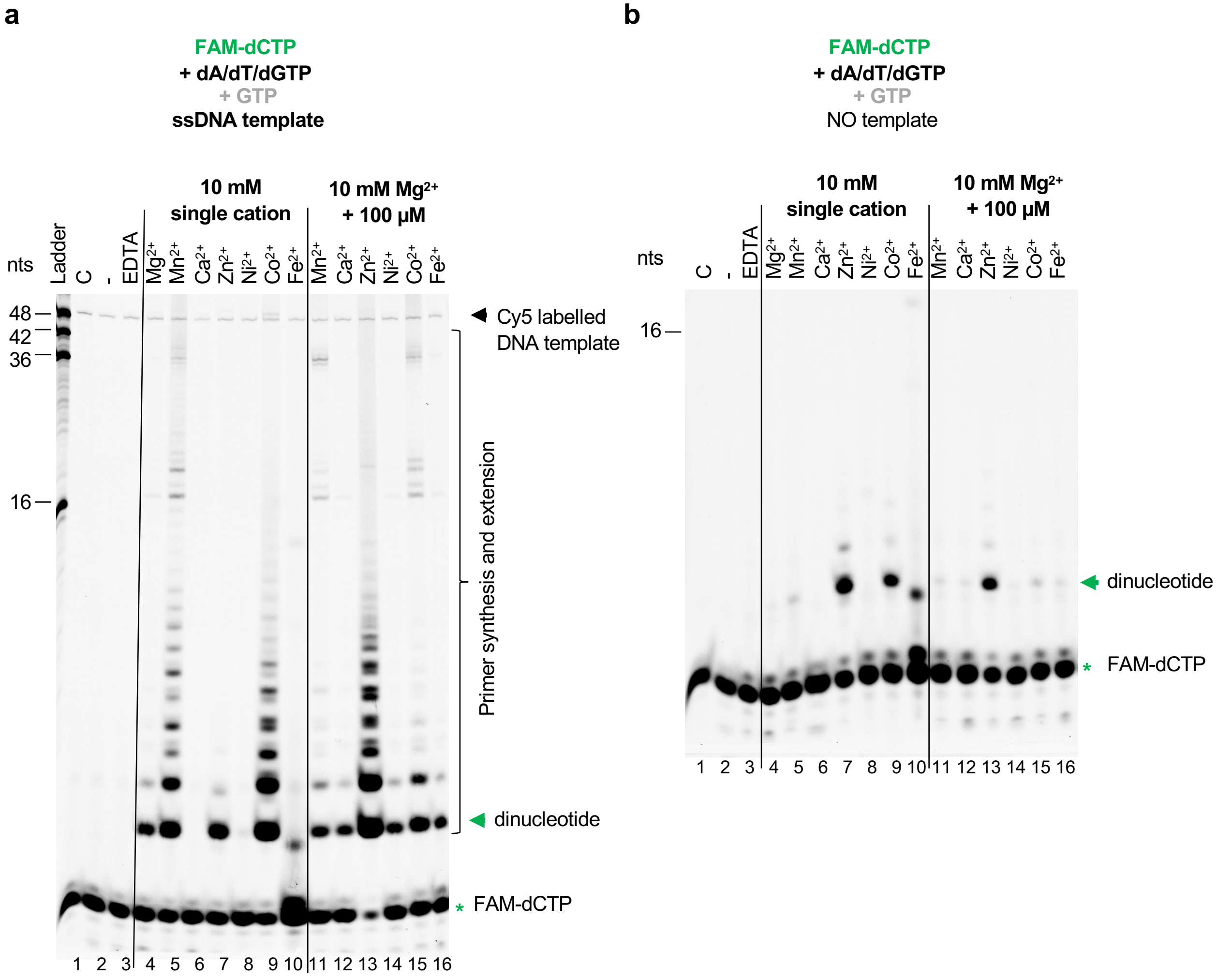

Supplementary Figure 6: Cation dependency of MpCAPP's DNA primase activity. (a) Cation dependency of MpCAPP's DNA primase activity in presence of ssDNA template. Lane 1 contained no MpCAPP (control: C), in the remaining lanes its concentration was 1 µM. Lane 2 (-) contained no cation. Lane 3 contained 10 mM EDTA. In lanes 4–10 the indicated cation (10 mM) was present. Lanes 11–16 contained 10 mM Mg^{2+} and another 100 µM cation, as indicated. All reactions contained 1 μM ssDNA template (oKZ53), 2.5 μM dNTPs (dCTP was FAM-labelled), 100 μM GTP and 10 mM Bis-Tris Propane; pH 7. Reactions were incubated at 50 °C for 30 min. Note: leakage of signal of Cy5-labelled DNA template is indicated by black arrow. Oligonucleotide (nts) length marker is shown on the left of the gel. **(b)** Cation dependency of MpCAPP's DNA primase activity in reaction without template. The experiment was performed in the same set up as above (panel a) containing different combinations of cations, 1 µM MpCAPP, 2.5 μM dNTPs and 100 μM GTP with no template DNA. Green arrow: dinucleotide, Green star: free FAM-labelled dCTP. Results shown representative of three independent repeats (6a,b).

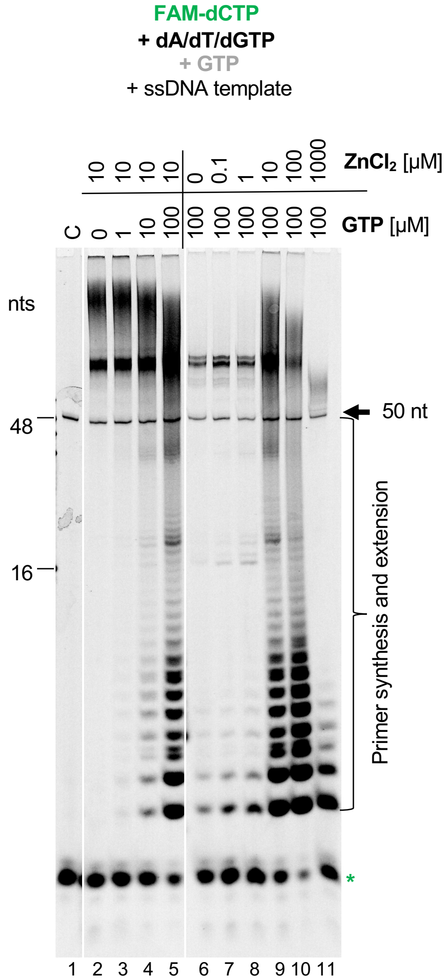

Supplementary Figure 7: GTP and Zn^{2+} Stimulate MpCAPP priming. Increasing concentration of GTP ribonucleotide stimulates MpCAPP priming activity and the optimal concentration of Zn^{2+} is between 10–100 μM. The reaction contained 4 μM MpCAPP, 1 μM DNA template (oKZ53), 2.5 μM dNTPs (dCTP was FAM-labelled) and increasing concentration of GTP from 0 to 100 μM (left) in presence of buffer containing 10 μM Bis-Tris Propane; pH 7, 10 mM $MgCl_2$ and 10 μM $ZnCl_2$. Or the concentration of the GTP was kept constant (100 μM) and the concentration of Zn^{2+} was changed as indicated (right). All reactions were incubated at 50 °C for 30 min. C: control reaction without protein, Black arrow: signal of Cy5-labelled template, Green star: FAM-dCTP mononucleotide. Oligonucleotide (nts) length marker is shown on the left of the gel. Results shown representative of three independent repeats.

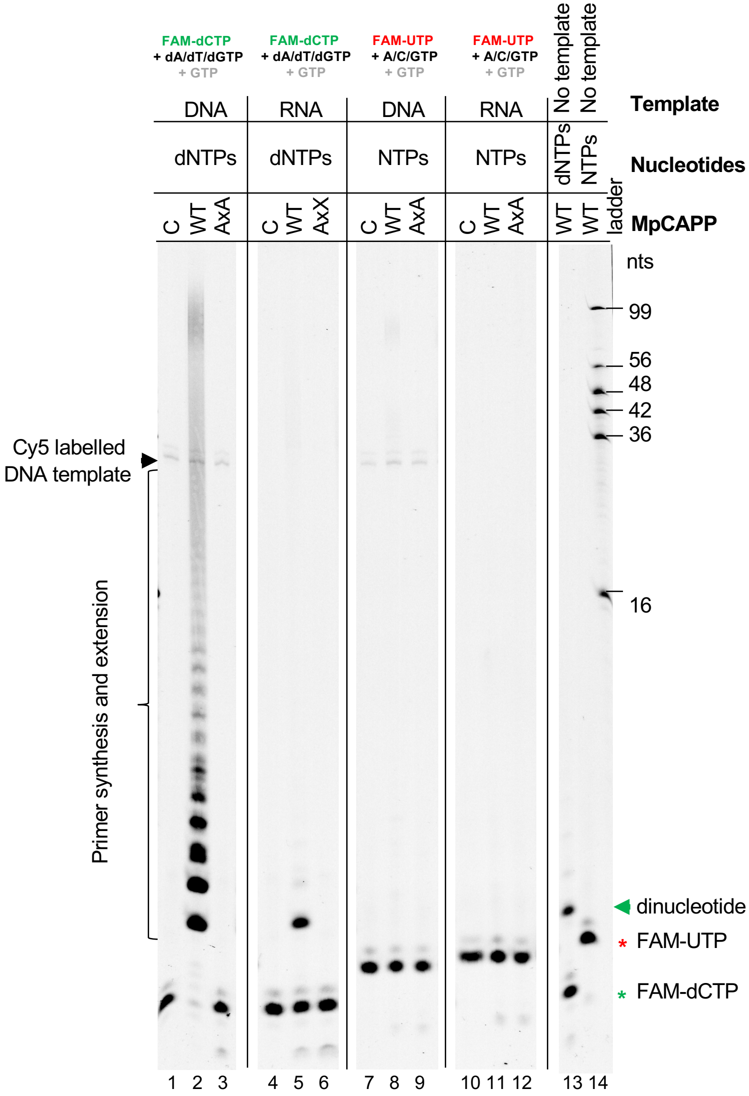

Supplementary Figure 8: MpCAPP is DNA-dependent DNA primase. The primase assay contained no enzyme (control: C), 1 µM MpCAPP WT or its AxA mutant. Lanes 1–3 contained 1 µM DNA template (oKZ116), 2.5 µM dNTPs (dCTP was FAM-labelled). Lanes 4–6 contained 1 µM DNA template (oKZ116) and 2.5 µM NTPs (UTP was FAM-labelled). Lanes 7–9 contained 1 µM RNA template (oKZ151), 2.5 µM dNTPs (dCTP was FAM-labelled). Lanes 10–12 contained RNA template (oKZ151) and 2.5 µM NTPs (UTP was FAM-labelled). Lane 13 contained no template, 2.5 µM dNTPs (dCTP was FAM-labelled) and lanes 14 contained no template and 2.5 µM NTPs (UTP was FAM-labelled). All reactions were performed in presence of 100 µM GTP and MpPrimBuffer and incubated at 50 °C for 30 min. Note: leakage of signal of Cy5-labelled DNA template is indicated by black arrow. Green arrow: dinucleotide, Green star: free FAM-dCTP, Red star: free FAM-UTP. Oligonucleotide (nts) length marker is shown on the left of the gel. Results shown representative of three independent repeats.

a

Templates:

oKZ152: 3′ T_{18}TCATT_{18}-p 5′
oKZ294: 3′ T_{18}TCTTT_{18}-p 5′
oKZ153: 3′ T_{18}TCCTT_{18}-p 5′
oKZ155: 3′ T_{18}TCGTT_{18}-p 5′
oMZ1 : 3′ T_{18}ACCTT_{18}-p 5′
oMZ2 : 3′ T_{18}CCCTT_{18}-p 5′
oKZ295: 3′ T_{18}GCCTT_{18}-p 5′
oKZ171: 3′ T_{18}TCCAT_{18}-p 5′
oKZ300: 3′ T_{18}TCCGT_{18}-p 5′

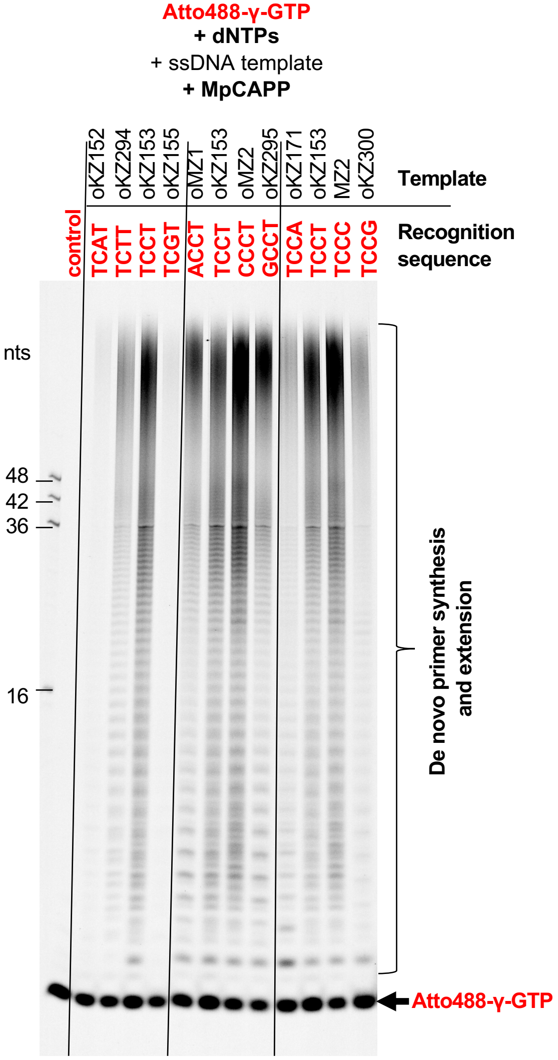

b

Templates:

oKZ152: 3′ T_{18}TCATT_{18}-p 5′
oKZ153: 3′ T_{18}TCCTT_{18}-p 5′
oKZ155: 3′ T_{18}TCGTT_{18}-p 5′
oKZ294: 3′ T_{18}TCTTT_{18}-p 5′
oKZ171: 3′ T_{18}TCCAT_{18}-p 5′
oMZ1 : 3′ T_{18}ACCTT_{18}-p 5′
oMZ2 : 3′ T_{18}CCCTT_{18}-p 5′
oMZ3 : 3′ T_{18}CCCCT_{18}-p 5′
oKZ241: 3′ T_{17}AACCT_{19}-p 5′
oKZ295: 3′ T_{18}GCCTT_{18}-p 5′
oKZ298: 3′ T_{18}ACCAT_{18}-p 5′
oKZ299: 3′ T_{18}GCCGT_{18}-p 5′
oKZ300: 3′ T_{18}TCCGT_{18}-p 5′
oKZ301: 3′ T_{18}ACCGT_{18}-p 5′
oKZ296: 3′ T_{18}GCCAT_{18}-p 5′

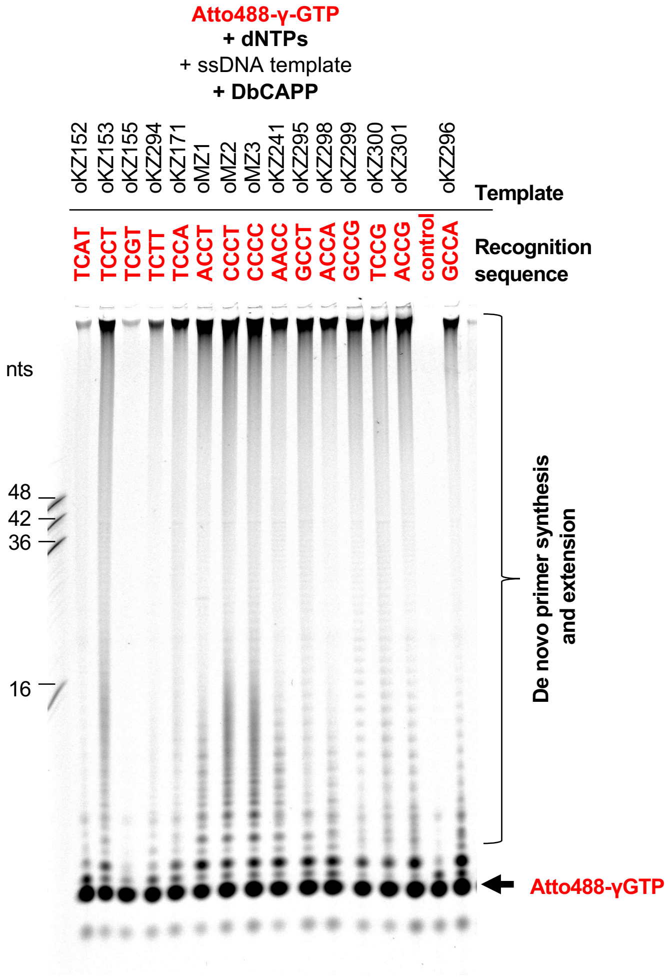

Supplementary Figure 9

Supplementary Figure 9: CAPP preferred template sequence for initiation of priming is 3'-XCCX-5'. (a) Test of different MpCAPP priming recognition sequences. Top: List of tested ssDNA templates. Bottom: 1 μM of MpCAPP (C-terminal MBP) was added into the reaction containing 1 μM DNA substrate as indicated, 100 μM dNTP mix and 10 μM γ-phosphate Atto488-labelled GTP. The reactions were incubated at 50 °C for 30 min. The products were resolved on 25 % urea-PAGE gel. Control: reaction without template. Oligonucleotide (nts) length marker is shown on the left of the gel. **(b)** Test of different DbCAPP priming recognition sequences. Top: List of tested ssDNA templates. Bottom: 1 μM of DbCAPP (C-terminal MBP) was added into the reaction containing 1 μM DNA substrate as indicated, 100 μM dNTP mix and 10 μM γ-phosphate Atto488-labelled GTP. The reactions were incubated at 50 °C for 30 min. The products were resolved on 20 % urea-PAGE gel. Control: reaction without template. Oligonucleotide (nts) length marker is shown on the left of the gel. Results shown representative of three independent repeats (9a, b).

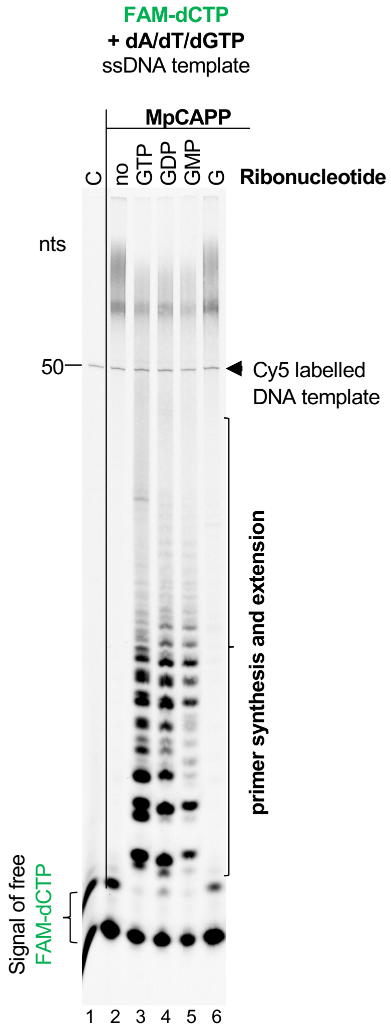

Supplementary Figure 10: MpCAPP prefers GTP before GDP or GMP for priming. Test of MpCAPP priming in presence of GTP, GDP GMP or G. 1 μM of MpCAPP WT protein was added into the reaction containing 1 μM ssDNA random sequence template (oKZ53), 2.5 μM non-labelled dATP, dTTP, dGTP, 2.5 μM FAM-dCTP and 100 μM GTP, GDP, GMP or G. The reactions were incubated at 50 °C for 30 min. The products were resolved on 20 % urea-PAGE gel. C: control reaction without protein. Oligonucleotide (nts) length marker is shown on the left of the gel. Results shown representative of three independent repeats.

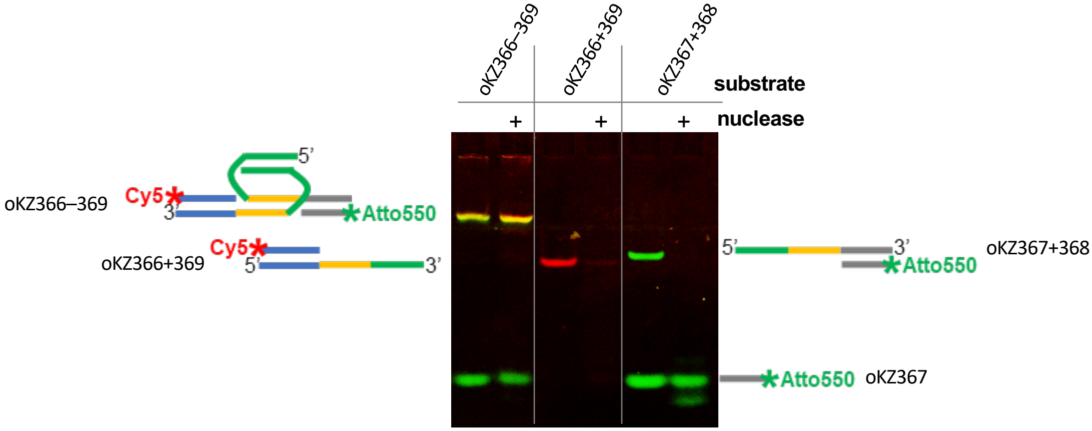

Supplementary Figure 11: Quality test of the post-synaptic DNA substrate used in displacement assay. 30 nM substrates were incubated with or without 1 U of Mung Bean Nuclease (NEB) for 10 min. at 37 °C and resolved on 1x TBE 8 % native PAGE gel. Results shown representative of two independent repeats.

a

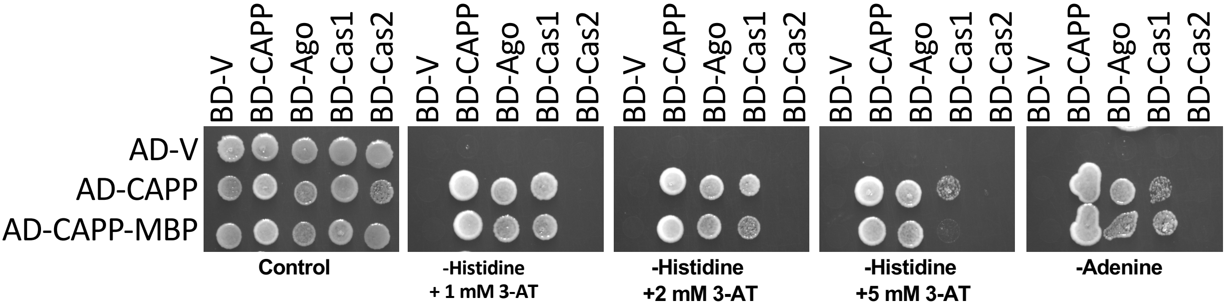

b

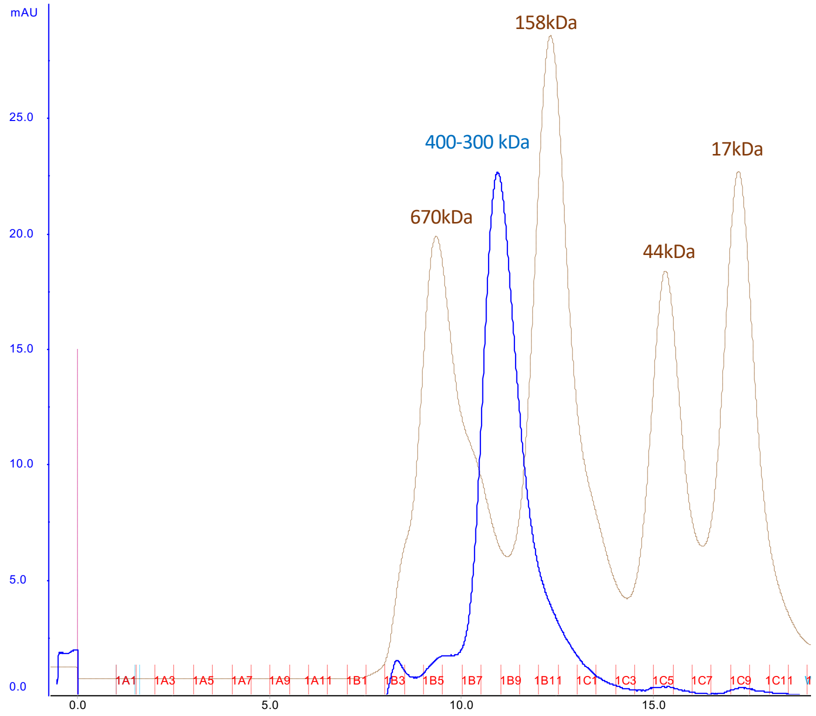

Supplementary Figure 12: MpCAPP-MBP fusion has only subtle effect on MpCAPP protein-protein interactions and forms a multimeric complex. (a) Comparison of interactions of MpCAPP and MpCAPP-MBP with MpCAPP, MpAgo and MpCas1 in the yeast two–hybrid assay. GAL4 DNA-binding domain (BD); activation domain (AD); empty vector (V). Interactions were established on selective plates lacking leucine, tryptophan, histidine or adenine. Addition of 3-amino-triazole (3-AT) was also used to increase stringency of the HIS3 reporter. Results shown representative of three independent repeats. **(b)** Analytical size exclusion chromatography (Cytiva, Superdex 200 10/300) of MpCAPP-MBP. Brown: protein standard, Blue: MpCAPP-MBP.

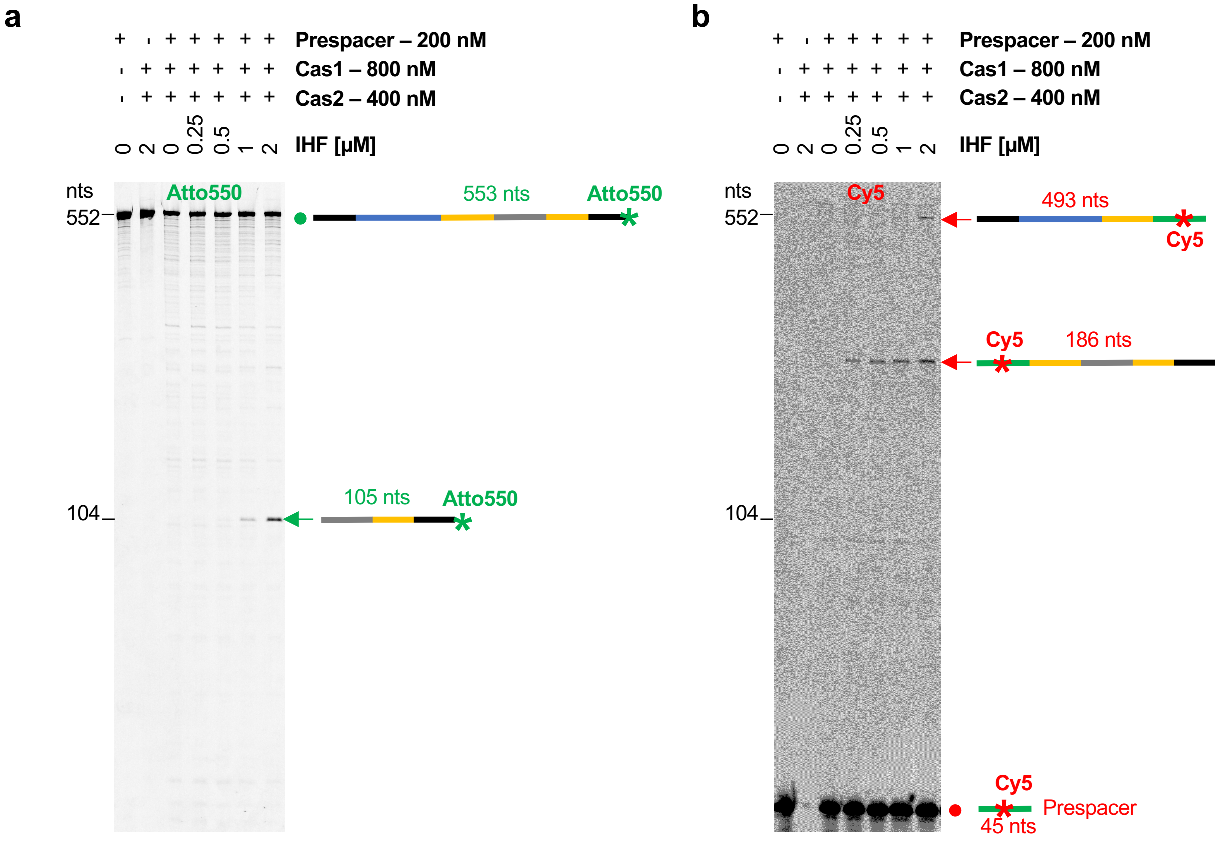

Supplementary Figure 13: Cas1 site-specific integration is directed by MpIHF. Split Atto550 **(a)** and Cy5 **(b)** channels of Figure 7b. For details, see Figure 7a and 7b. Results shown representative of three independent repeats (13a, b).

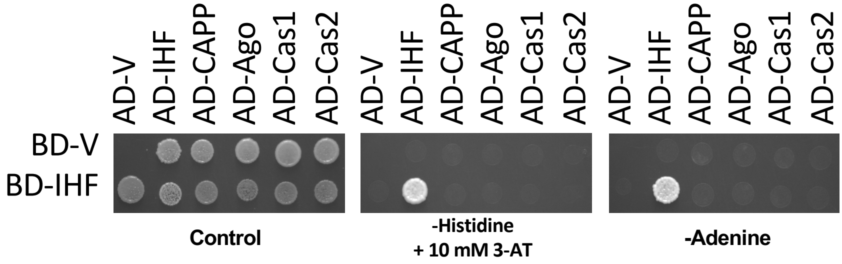

Supplementary Figure 14: MpIHF self-associate. Y2H Test of interactions of MpIHF with MpIHF, MpCAPP, MpAgo and MpCas1. GAL4 DNA-binding domain (BD); activation domain (AD); empty vector (V). Interactions were established on selective plates lacking leucine, tryptophan, histidine or adenine. Addition of 3-amino-triazole (3-AT) was also used to increase stringency of the HIS3 reporter. Results shown representative of three independent repeats.

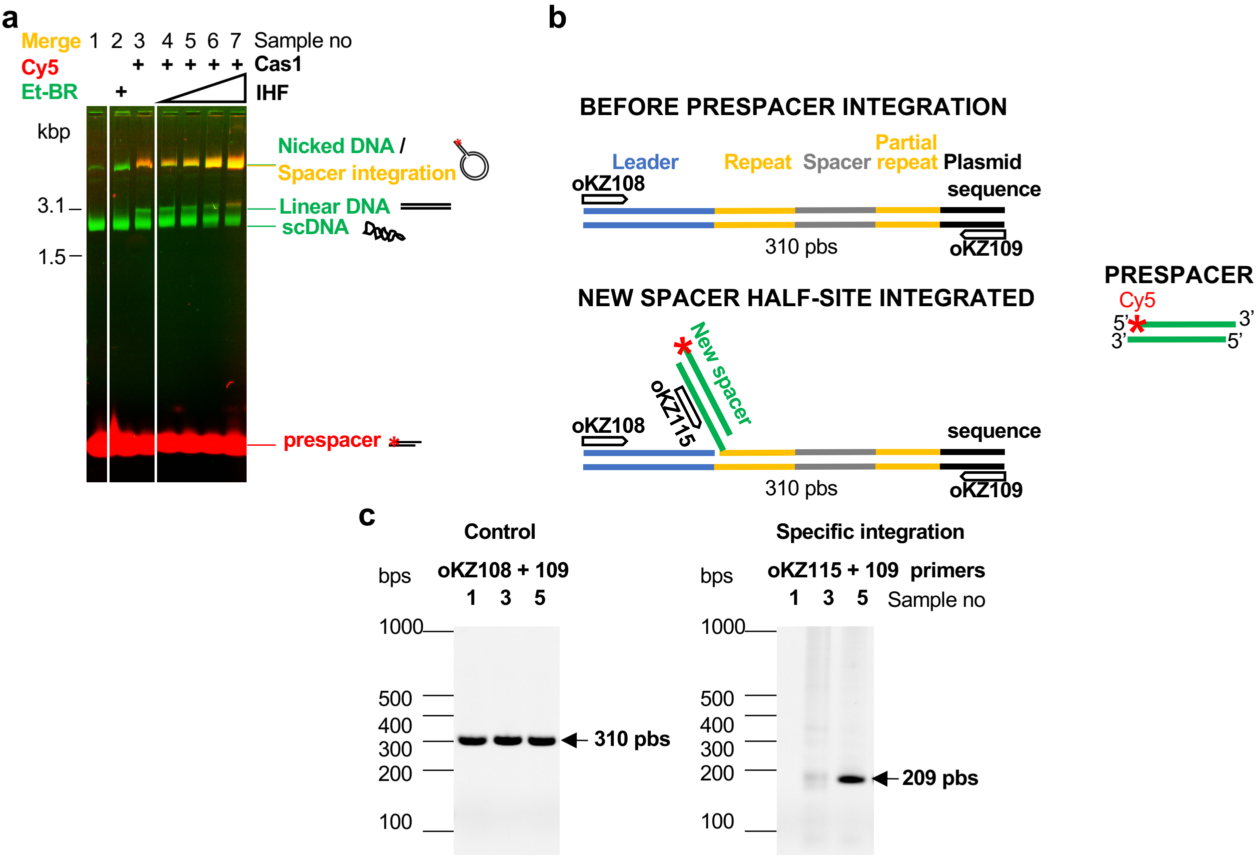

Supplementary Figure 15: Cas1 site-specific half-site integration is directed by MpIHF. (a) CRISPR array-containing plasmid (7.9 nM) was resolved on agarose gel after MpCas1 (400 nM) prespacer integration in presence of increasing concentration if IHF in buffer containing 20 mM Tris.HCl; pH 7.5, 10 mM NaCl, 1 mM DTT and 10 mM $MgCl_2$. The reaction was incubated for 1 hour at 50 °C before phenol-chloroform-isoamyl alcohol purification and loaded on the agarose gel. Green signal: Et-Br, Red signal: Cy5 (prespacer), Yellow signal: overlapping green and red signal. Results shown representative of three independent repeats. **(b)** Schematic representation of CRISPR array before and after prespacer integration. White arrows show primer (oKZ108, oKZ109 and oKZ115) direction used in PCR (panel c). **(c)** PCR shows MpCas1 CRISPR array-targeted integration of prespacer in presence of MpIHF. Control PCR with 310 bp product shows, that the amount of the plasmid DNA is in all reactions comparable. Specific integration PCR with 220 bp product shows that the MpCas1 CRISPR-array directed prespacer integration is stimulated in presence of MpIHF.

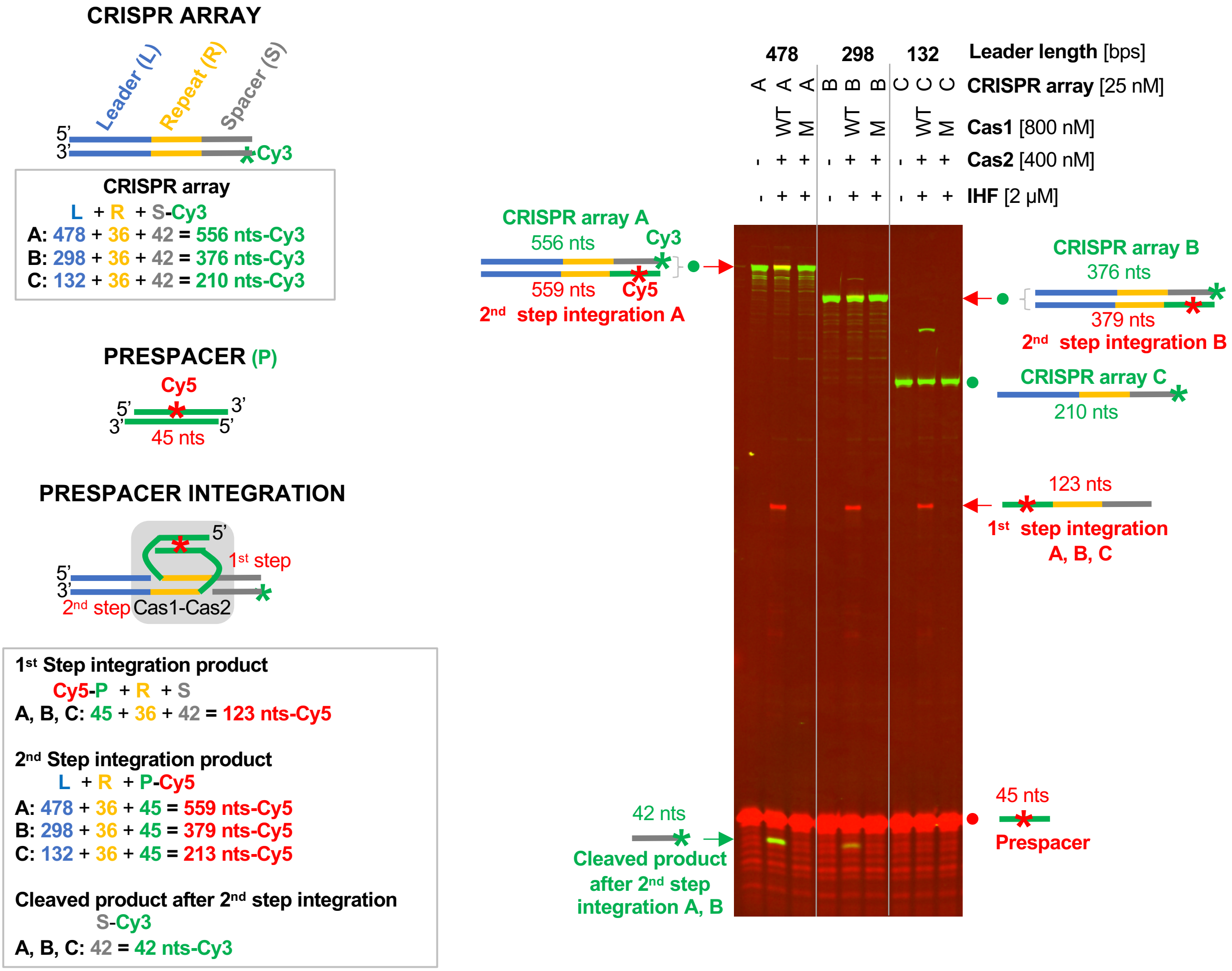

Supplementary Figure 16: MpCas1 integration activity shows leader length dependency. Schematic representation of CRISPR array (left) before and after prespacer integration used in the Cas1-integration assay (right). CRISPR arrays A, B and C (see Supplementary Note3) with 3 different leader lengths (478, 298 and 132 bps, respectively) were incubated with wild-type MpCas1 (WT) protein or its H223A mutant (M) in presence of MpCas2, MpIHF and 200 nM prespacer (Cy5-labelled) in buffer containing 10 mM Bis-Tris Propane; pH 7, 10 mM MgCl2, 100 mM NaCl, 0.1 mg/ml BSA and 0.5 mM TCEP for 90 min. at 50 °C. After Proteinase K digestion the products were resolved on denaturing Urea-PAGE. Green signal: Cy3, Red signal: Cy5, Yellow signal: overlapping green and red signal, Red dot: prespacer, Green dot: CRISPR array without any integration, Red and green arrows: product after prespacer integration. Results shown representative of three independent repeats.

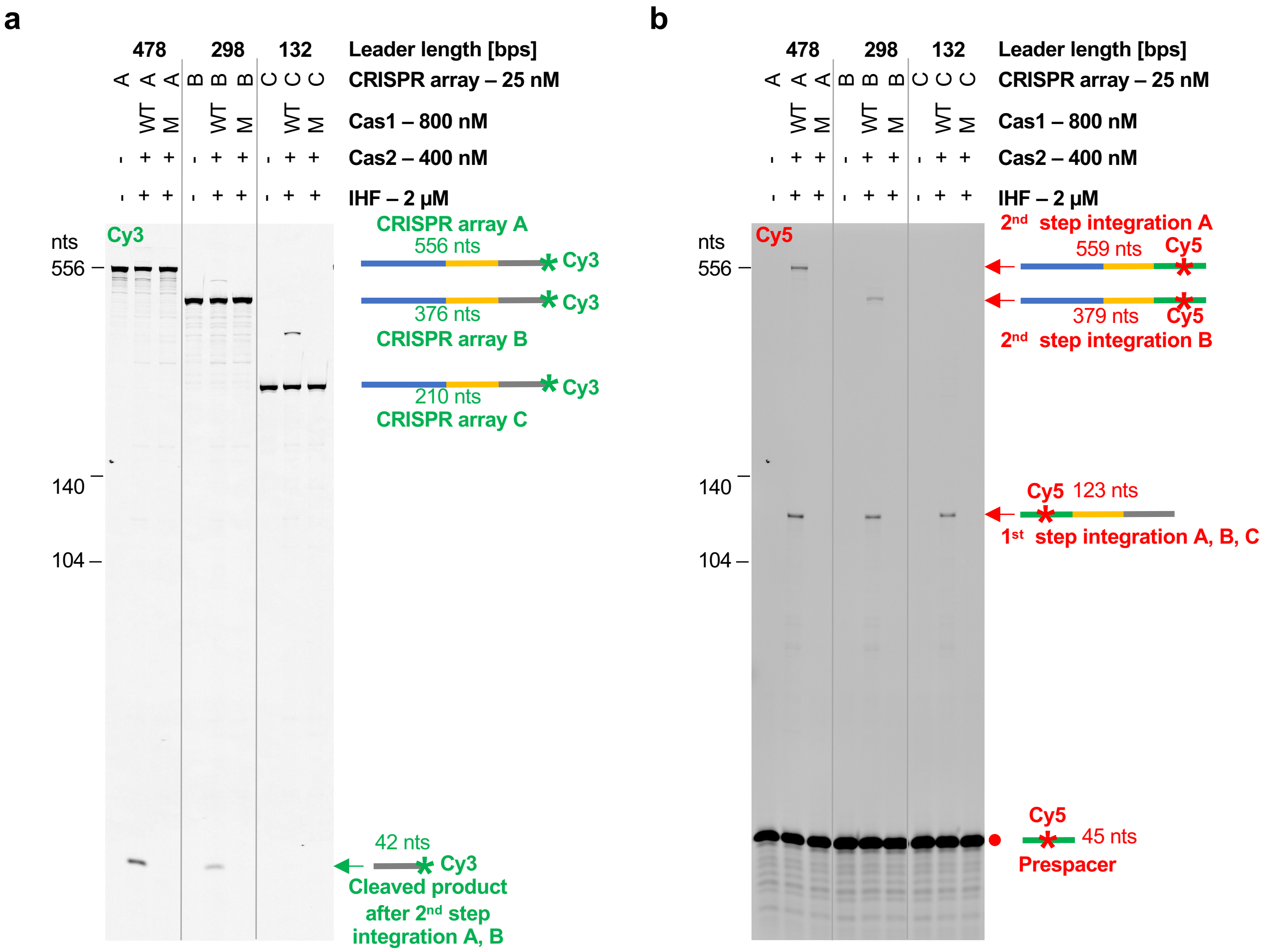

Supplementary Figure 17: Leader length dependency of MpCas1 integration activity. Split Cy3 **(a)** and Cy5 **(b)** channels of Supplementary Fig. 16 (right). For details, see Supplementary Fig. 16. Results shown representative of three independent repeats (17a, b).

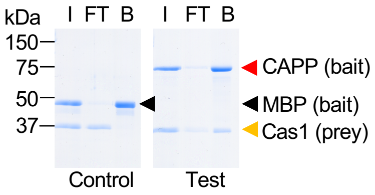

Supplementary Figure 18: DbCAPP interacts with DbCas1 in pull-down assay. Bait DbCAPP (C-terminal MBP) or MBP was pre-bound to amylose beads and washed before adding of prey DbCas1 (see Supplementary methods) similarly as pull-downs for MpCAPP. I: combined sample of bait and prey input, FT: flow-through, B: bound protein. The fractions were resolved on SDS-PAGE and Coomassie stained. Results shown representative of three independent repeats.

Fig. 5c

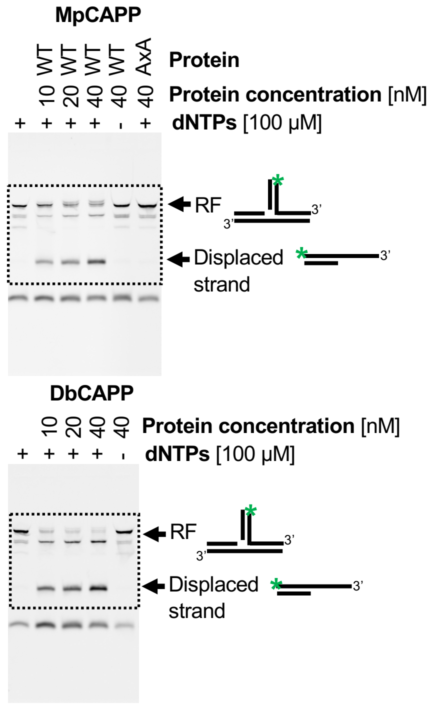

Fig. 8c

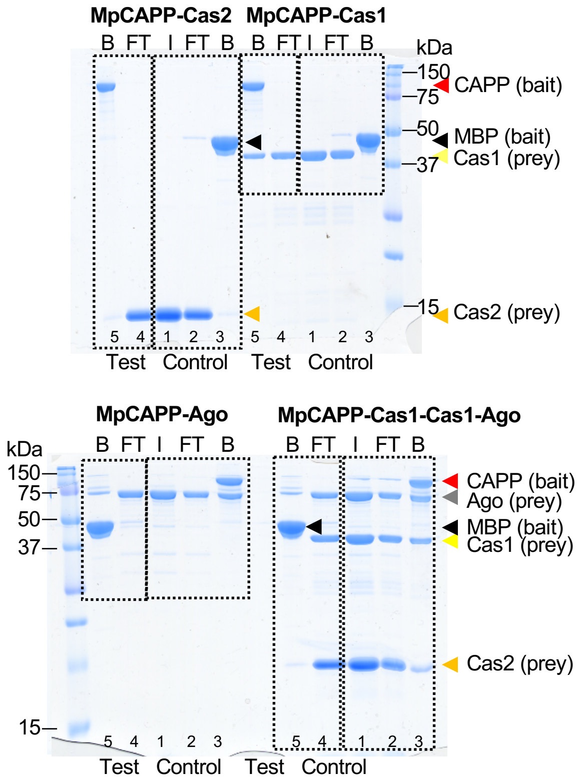

Suppl. Fig. 14c

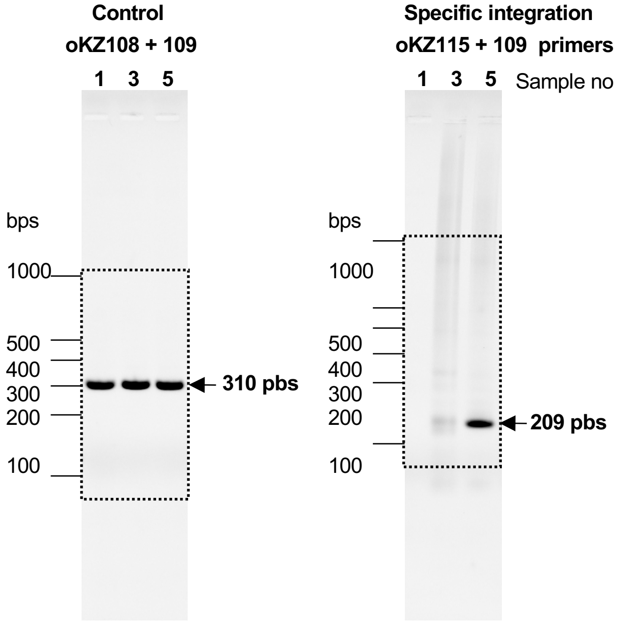

Suppl. Fig. 17

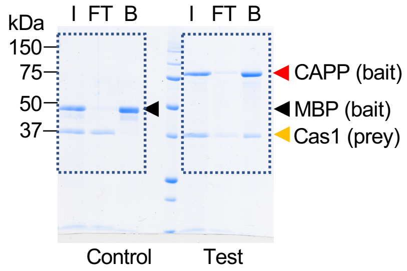

Supplementary Figure 19: Images of the uncropped gel used in this study. Cropped regions from these gels used in the presented figures are shown as hatched boxes.

Supplementary Methods

Plasmid based *in vitro* MpCas1 prespacer integration assay

The assay was performed essentially as described here[38] with some modifications. The reaction was performed in 20 μl. First, 400 nM MpCas1 and 100 nM prespacer (duplex of oKZ52 and oKZ104 oligonucleotides) were incubated together for 30 min. at 37 °C in reaction buffer (20 mM Tris.HCl; pH 7.5, 10 mM NaCl, 1 mM DTT, 10 mM $MgCl_2$) before adding 7.9 nM MpCRISPR array-containing plasmid pKZ73 (Supplementary Note 1 (top CRISPR array)) and increasing concentration of the MpIHF protein. The reaction was further incubated for 60 min. at 50 °C. The reaction was stopped by adding of 0.8 U Proteinase K (NEB) and 0.4 μl of 0.5 M EDTA and incubated for 30 min. 37 °C followed by Phenol-chloroform-isoamyl alcohol purification (20 μl of Phenol-chloroform-isoamyl alcohol (25 : 24 : 1); pH 8 was added into each reaction, vortexed and centrifuged for 10 min. at 16, 000 g. 20 μl of the aqueous phase was added to the 15 μl of chloroform-isoamyl alcohol (24 : 1) vortexed and centrifuged for 10 min. at 16, 000 g). 17 μl of the aqueous phase was mixed with 4 μl of 24% Ficoll 400 and run on 1.8 % TAE agarose gel containing ethidium bromide for 90 min. at 120 V. For PCR, 1 μl of the aqueous phase was diluted with 9 μl of H_2O. 0.5 μl of the diluted DNA product was used in 10 μl PCR reaction containing Q5 High Fidelity DNA polymerase (NEB). The NEB standard Q5 High Fidelity DNA polymerase protocol was used (Ta = 60 °C, Extension time = 1 min., 25 cycles) using combination of primers oKZ108 and oKZ109 as a control PCR and oKZ115 and oKZ109 for testing of the specific CRISPR-array integration. The PCR products were resolved on 1.5 % TAE agarose gel containing ethidium bromide.

Synthesis of substrates *for in vitro* MpCas1-Cas2 prespacer integration assay on DNA fragments

M. piezophila CRISPR arrays were synthesized by PCR (Q5 High Fidelity DNA polymerase, NEB). CRISPR array A: template – pKZ137, primers: oKZ355 and oKZ356, CRISPR array B: template – pKZ137, primers: oKZ357 and oKZ356, CRISPR array C: template – pKZ137, primers: oKZ304 and oKZ356 (Supplementary Note 1 (CRISPR arrays A–C)). All substrates (PCR products) were resolved on 2 % agarose gel and gel-extracted before use in the assays. The assay is further described in main methods.

DbCas1 expression and purification

DbCas1 protein was expressed from pMZ114 in BL21(DE3) in TB media supplemented with trace metals and induced with 1 mM IPTG at 37 °C for 3 h. DbCas1 purification: Cell paste was resuspended in 50 mM Tris pH 7.5, 0.15 M NaCl, 20 % glycerol, 30 mM imidazole, 0.5 mM TCEP, sonicated and cleared by ultracentrifugation. The supernatant was loaded on HisTrap column (Cytiva) and eluted in the same buffer with gradient to 0.5 M imidazole. Major peak was collected and loaded onto StreptactinXT HC (IBA) and eluted with the same buffer with 50 mM biotin added. The Strep-tagged DbCas2 was found insoluble, therefore the His-tagged DbCas1 in the flow-through was concentrated in Vivaspin 20 (Sartorius) and resuspended to 50 % glycerol, aliquoted, frozen in liquid nitrogen and stored at −80 °C.

Supplementary Note 1

Sequences of tested CRISPR arrays

CRISPR array (used in Fig. 7 and Suppl. Fig. 14)

AAAGATATTCGCAAAATAGTCAAAATTATAAAATACGCATAAATAAACCAAAACATATCTACGAGGTTCGCAG
AGGTTTATGATTTTGTAACACTTTTCAAAAACCACTTGACAAAAGGATTTTAGAATGTTATAATAAAAATAGTT
CGTATACGGACCTAAATTCAAAAACACAAAATCTCTGAAAGCAGCTTAAACAAAAGAAAACTGAAAAACAGG
TACGAATATAGATGAAAAGCGGATTACAGAGATTTTATTCGAAAAAATGCCGAAAATTTCAAAATTAAAAAAC
AGGTTCGAAAAATCACCTTCTAAAAAACGCTAATAATAACCATTCTAGAAACCAGCTATCCGAATATCACTACT
CTCTGAAAGAGATAGAAACAATCCCATTCAACGTTTAATACTTTTTTAAATAACCAATTATATCCGAATATCACT
ACTCTCT-Atto550

CRISPR array A

AATTTCCAGCCATCTACTCTTTACGATGGCTGGTTTTTTATATATAAAATAACTGATTTTTTGATATAAGTTGCA
ATTACATTTAATAAAAGATTTATAAAAAAACTAAATATAAAAGAAAAACAAAAGAAAGATATTCGCAAAATAG
TCAAAATTATAAAATACGCATAAATAAACCAAAACATATCTACGAGGTTCGCAGAGGTTTATGATTTTGTAACA
CTTTTCAAAAACCACTTGACAAAAGGATTTTAGAATGTTATAATAAAAATAGTTCGTATACGGACCTAAATTCA
AAAACACAAAATCTCTGAAAGCAGCTTAAACAAAAGAAAACTGAAAAACAGGTACGAATATAGATGAAAAGC
GGATTACAGAGATTTTATTCGAAAAAATGCCGAAAATTTCAAAATTAAAAAACAGGTTCGAAAAATCACCTTCT
AAAAAACGCTAATAATAACCATTCTAGAAACCAGCTATCCGAATATCACTACTCTCTGAAAGAGATAGAAACA
ATCCCATTCAACGTTTAATACTTTTTTAAATAACCAATTAT-Cy3

CRISPR array B

AACATATCTACGAGGTTCGCAGAGGTTTATGATTTTGTAACACTTTTCAAAAACCACTTGACAAAAGGATTTTA
GAATGTTATAATAAAAATAGTTCGTATACGGACCTAAATTCAAAAACACAAAATCTCTGAAAGCAGCTTAAAC
AAAAGAAAACTGAAAAACAGGTACGAATATAGATGAAAAGCGGATTACAGAGATTTTATTCGAAAAAATGCC
GAAAATTTCAAAATTAAAAAACAGGTTCGAAAAATCACCTTCTAAAAAACGCTAATAATAACCATTCTAGAAAC
CAGCTATCCGAATATCACTACTCTCTGAAAGAGATAGAAACAATCCCATTCAACGTTTAATACTTTTTTAAATAA
CCAATTAT-Cy3

CRISPR array C

GGTACGAATATAGATGAAAAGCGGATTACAGAGATTTTATTCGAAAAAATGCCGAAAATTTCAAAATTAAAAA
ACAGGTTCGAAAAATCACCTTCTAAAAAACGCTAATAATAACCATTCTAGAAACCAGCTATCCGAATATCACTA
CTCTCTGAAAGAGATAGAAACAATCCCATTCAACGTTTAATACTTTTTTAAATAACCAATTAT-Cy3

Leader
Repeat
Spacer

Supplementary Note 2

Marinitoga piezophila CRISPR arrays

>CRISPR1 Leader (found between Marpi_0401 and Marpi_0401 (MpCAPP))

AATTTCCAGCCATCTACTCTTTACGATGGCTGGTTTTTTATATATAAAATAACTGATTTTT
TGATATAAGTTGCAATTACATTTAATAAAAGATTTATAAAAAAACTAAATATAAAAGAAAA
ACAAAAGAAAGATATTCGCAAAATAGTCAAAATTATAAAATACGCATAAATAAACCAAAA
CATATCTACGAGGTTCGCAGAGGTTTATGATTTTGTAACACTTTTCAAAAACCACTTGAC
AAAAGGATTTTAGAATGTTATAATAAAAATAGTTCGTATACGGACCTAAATTCAAAAACAC
AAAATCTCTGAAAGCAGCTTAAACAAAAGAAAACTGAAAAACAGGTACGAATATAGATGA
AAAGCGGATTACAGAGATTTTATTCGAAAAAATGCCGAAAATTTCAAAATTAAAAAACAG
GTTCGAAAAATCACCTTCTAAAAAACGCTAATAATAACCATTCTAGAAACCAGCT

>CRISPR2 Leader (found between Marpi_0414 (MpCmr1) and Marpi_0415 (AAA-ATPase))

TTTTTCCCTCCTATTTATTAAAAATTAACATCTTCAACCAATCAATGATATATTAATAATAT
AAATATATTCTAATTAATCTTGAATTAAATTATACATTTCTATTTTAACACATCACAAAATT
AATTTCAAGAAAATATAAGACCCCTGAAATTAATTGTAAAGGTTAAAGTAGAAAAGAATA
AAAAGTGTGTATATGGTAAAACAAATTGACCCCTCTAAGGAAGAAAAAGCTTATGATGA
GTAGTAAAATAGAAATATAACTCGTCGAAAGGAGAATGGGGGATGAAAATAATAATGGA
AGCATATGAGATTATTACAATGCACAAGGAAATATAAGCAATAATAAAGAATGGATAAAA
AAAACAGCAATACTCGGAAATAAGAAATTTATAAATAAACTAAATATAAAAGAAAAACAAA
AGAAAGATATTCGCAAAAATAACAAAACTATAAAATACGCATAAATAAACTAAAACATATC
TATGAGGTTCGCAGAGGTTTATAATTTTGTAACACTTTTCAAAAATCACTTGACAAAAGG
ATTTTAGAATGTTATAATAGAAATAGTTCGTATACGGACCTAAATCCAAAAACACAAAATC
TCTGAAAGCGGCTTAAACAAAGGAAAACTGAAAAACAGGTACGAATATAGATAAAAAGC
AGATTATAGAGATTTTATTCGAAAAAATGCTGAAAATTTCAAAATTAAAAAACAGGTTCGA
AAAATCACCTTCTAAAAAACGCTAATAATAACCATTCTAGAAACCAGCT

>CRISPR3 Leader (found between Marpi_0421 and Marpi_0422)

GACTTTTTGTATACTCTCCTTCAAAATAGAGGGAGAGTATTGTATATGGTAAAATAAATC
GCCCCCTCTAAGGAAGAAAAAGCTTATGATGAGTAGTAAAATAGAAATATAACTCGTCG
AAAGGAGAATGGGGGATGAAAACAATAATGGAAGCATATGAGATTATTACAATGTACAA
GGGAATATAAGCAATAATAAAGAATGGATAAAAAAAACAGCAATATTCGGAAATAAGAAA
TTTATAAATAAACTAAATATAAAAGAAAAACAAAAGAAAGATATCCGCAAAAATAACAAAA
CCATAAAATACGCATAAATAAACTAAAACATATCTATGAGGTTCGCAGAGGTTTATGATT
TTGTAATACTTTTCAAAAATCACTTGACAAAAGGATTTTAGAATGTTATAATAAAAATAGT
TCGTATACGGACCTAAATCCAAAAACACAAAATCTCTGAAAGCGGCTTAAACAAAGAAA
AACTGAAAAACAGGTACGAATATAGATAAAAAGCCGATTATAGAGATTTTATTCGAAAAA
ATGCTGAAAATTTCAAAATTAAAAAACAGGTTCGAAAAATCACCTTCTAAAAAACGCTAA
TAATAACCATTCTAGAAACCAGCT

Supplementary Note 3

Sequence alignment of *Marinitoga piezophila* CRISPR arrays

```
CRISPR1         ------------------------------------------------------------   0
CRISPR2         TTTTTCCCTCCTATTTATTAAAAATTAACATCTTCAACCAATCAATGATATATTAATAAT   60
CRISPR3         ------------------------------------------------------------   0


CRISPR1         ------------------------------------------------------------   0
CRISPR2         ATAAATATATTCTAATTAATCTTGAATTAAATTATACATTTCTATTTTAACACATCACAA   120
CRISPR3         ------------------------------------------------------------   0


CRISPR1         ------------------------------------------------------------   0
CRISPR2         AATTAATTTCAAGAAAATATAAGACCCCTGAAATTAATTGTAAAGGTTAAAGTAGAAAAG   180
CRISPR3         -------------------------------GACTTTTTGTATACTCTCCTTCAAAATAG   29


CRISPR1         ------------------------------------------------------------   0
CRISPR2         AATAAAAAGTGTGTATATGGTAAAACAAATTGACCCCTCTAAGGAAGAAAAAGCTTATGA   240
CRISPR3         AGGGAGAGTATTGTATATGGTAAAATAAATCGCCCCCTCTAAGGAAGAAAAAGCTTATGA   89

                                                  CRISPR array A →
CRISPR1         --------------------------------------------------------AATT   4
CRISPR2         TGAGTAGTAAAATAGAAATATAACTCGTCGAAAGGAGAATGGGGGATGAAAATAATAATG   300
CRISPR3         TGAGTAGTAAAATAGAAATATAACTCGTCGAAAGGAGAATGGGGGATGAAAACAATAATG   149
                                                                        ***

CRISPR1         TCCAGCCATCTACTCTTTACGATGGCTGGTTTTTTATATATAAAATAACTGATTTTTTGA   64
CRISPR2         GAAGCATATGAGATTATTACAATGCACAAGGAAATATAAGCAATAATAAAGAATGGATAA   360
CRISPR3         GAAGCATATGAGATTATTACAATGTACAAGGGAATATAAGCAATAATAAAGAATGGATAA   209
                       **    *  **** ***          ****   ** *  *  ** *   * *

CRISPR1         TATAAGTTGCAATTACATTTAATAAAAGATTTATAAAAAAACTAAATATAAAAGAAAAAC   124
CRISPR2         AAAAAACAGCAATACTCGGAAATAAGAAATTTATAAATAAACTAAATATAAAAGAAAAAC   420
CRISPR3         AAAAAACAGCAATATTCGGAAATAAGAAATTTATAAATAAACTAAATATAAAAGAAAAAC   269
                 * **   *****       ***** * ********* **********************
    CRISPR array →                                CRISPR array B →
CRISPR1         AAAAGAAAGATATTCGCAAAATAGTCAAAATTATAAAATACGCATAAATAAACCAAAACA   184
CRISPR2         AAAAGAAAGATATTCGCAAAAATAACAAAACTATAAAATACGCATAAATAAACTAAAACA   480
CRISPR3         AAAAGAAAGATATCCGCAAAAATAACAAAACCATAAAATACGCATAAATAAACTAAAACA   329
                ************* *******    *****  ********************* ******

CRISPR1         TATCTACGAGGTTCGCAGAGGTTTATGATTTTGTAACACTTTTCAAAAACCACTTGACAA   244
CRISPR2         TATCTATGAGGTTCGCAGAGGTTTATAATTTTGTAACACTTTTCAAAAATCACTTGACAA   540
CRISPR3         TATCTATGAGGTTCGCAGAGGTTTATGATTTTGTAATACTTTTCAAAAATCACTTGACAA   389
                ****** ******************* ********* ************ **********

CRISPR1         AAGGATTTTAGAATGTTATAATAAAAATAGTTCGTATACGGACCTAAATTCAAAAACACA   304
CRISPR2         AAGGATTTTAGAATGTTATAATAGAAATAGTTCGTATACGGACCTAAATCCAAAAACACA   600
CRISPR3         AAGGATTTTAGAATGTTATAATAAAAATAGTTCGTATACGGACCTAAATCCAAAAACACA   449
                *********************** ************************* **********
                                      CRISPR array C →
CRISPR1         AAATCTCTGAAAGCAGCTTAAACAAAAGAAAACTGAAAAACAGGTACGAATATAGATGAA   364
CRISPR2         AAATCTCTGAAAGCGGCTTAAACAAAGGAAAACTGAAAAACAGGTACGAATATAGATAAA   660
CRISPR3         AAATCTCTGAAAGCGGCTTAAACAAAGAAAAACTGAAAAACAGGTACGAATATAGATAAA   509
                ************** ***********  ***************************** **

CRISPR1         AAGCGGATTACAGAGATTTTATTCGAAAAAATGCCGAAAATTTCAAAATTAAAAAACAGG   424
CRISPR2         AAGCAGATTATAGAGATTTTATTCGAAAAAATGCTGAAAATTTCAAAATTAAAAAACAGG   720
CRISPR3         AAGCCGATTATAGAGATTTTATTCGAAAAAATGCTGAAAATTTCAAAATTAAAAAACAGG   569
                **** ***** *********************** *************************

CRISPR1         TTCGAAAAATCACCTTCTAAAAAACGCTAATAATAACCATTCTAGAAACCAGCT 478
CRISPR2         TTCGAAAAATCACCTTCTAAAAAACGCTAATAATAACCATTCTAGAAACCAGCT 774
CRISPR3         TTCGAAAAATCACCTTCTAAAAAACGCTAATAATAACCATTCTAGAAACCAGCT 623
                ******************************************************
```

Supplementary Note 4

Protein coding sequences

MpCAPP (codon optimized)

ATGAACTTTAAAGAGCTGGCCTTTAAAGCCGAAGAGGATTTTGATTTTGAAAAAGCCATCGAGTATTA
CAAAAAAGCCTTTAACGAACTGACCATCAACGATGATAGCCTGATTCGTTATGCAAACCTGCTGTTTG
ATTTCCAGAAATTTGAAGAAGCCGAACCGATCTTTGAAAAAATCGTGAGCGAAATCAACGACAGCGAG
TATATGAGCAAACTGGCCATTATCTATGAAGAGAACAACAAATTTGAGAAAGCGCTGAAAATCTATAA
AGAACTGGGCATCGAAAGCAAGGTGAAAGAACTGAGCGATAAAATCGAACTGAAAGCACCGAGCCAGA
ATGCCATTAAACGTTTTATGACCCTGTTTAGCGGTCGCGAAGATGTTTTTAGCATTCAGTATGAAGGT
GGCTATCGTCCGATTCGTCGTCCGCTGAATTTTCATGATATCAAAGATCACTTCAGCGGCAAAAAAAC
CCTGGGTATTTATCTGCTGAAAAAGAACGATACCGTGAAATTCGCAGCCTATGATATCGACATCAAAA
AGCATTATCTGAACCGCGAGGATAAATTCGTGTATGAGGAAAACAGCAAAAAAGTTGCAAAACGTCTG
AGCCGTGAACTGAATCTGGAAAACATCATCCACTATTTTGAATTCACCGGCAATCGCGGTTATCACAT
CTGGATCTTTTTTGATATTCCGGTGAGCGCCTACAAGATCAAATACATCATGGAAAAAATCCTGGATC
GCATCGAACTGGAAGAGGGTATTGATGTGGAAATTTTTCCGAAACAGACCAGCCTGAATGGTGGTCTG
GGTAATCTGATTAAAGTTCCGCTGGGTGTTCACAAAAAAACGGGTAAAAAATGCCTGTTCGTGGACAA
CGATTTTAACGTGATTGAAAACCAGATCGAGTTCCTGAACAACATCAAAGAAAATAAAGCCACCGAGA
TCGATAAACTGTTTCGCGAGATTTTTAACGAGAACGATTATGACGATTTCAATGTGACCTACAAAAAA
CGTACCACGAGCAGCAAAAATAGCGCGAGCAAAAAAATCAAATCCAGCATTCGTAAAACCCTGCCGAA
AAGCAACAAAAACATTTTCACCCAGATGATCGAAGGCTGCCATATTCTGAAGCAGATCAACGAGAAAA
TCGAGAAAGAAGCCTATATCACGGAAGAGGAAGAAATGATCTTTATCAAAAGCCTGGCCAACCTGGAA
AATAGCAAGAAATTTATCGAGATGAAACTGAGCCAGACGATCAACTTTAGCAAAAAACGCATCGACAT
GATCATCAAACAGAGCCTGGGTGTGCCGATTACCTGTGAAGAAATCCGCAAAATCATTCTGAACAAAG
ATATTAGCCTGAGCCTGGAAAACTGTACCTGTAAATTTCAGGGTAACTATAATAGCCCGTATGCCATC
GTGGAAAATATCGAAGAGATGTTCCTGGACAAGATCGATATTAACGATATCGCCAAAAAGATTGTCGA
GAAAAACGCCGAAAAATTCGAAATCGAAAAAGAGATCAAAAACCTGAAACGCATGATCGCGAAAAAAA
TGGGTGATAGCAAAGAACTGCGTACCGAAATTGGTACGATTAAACGCTTTGGTGATGACATCGAAATC
ATCCTGTAATAA

MpCas2 (codon optimized)

ATGTTCTACGTTGTTTCTTACGACATCACCAACGACAAACGTCGTCGTAAAGTTGTTAAATACCTGGA
ATCTTACGGTGTTCGTGTTCAGTACTCTGTTTTCGAAACCGAACTGAACCAGGACCAGCTGAAAAAAC
TGATCAAAGGTCTGAAAAAACAGATCAAAAAAGACGAAGACACCATCCGTATCTACCCGATCTCTAAA
GAATCTCGTCGTTACATCGTTACCATCGGTATCGACAAAGGTAAATACTACGACAAAGACTTCCTGAT
CATCTAA

MpAgo (codon optimized – used for Y2H constructs)

ATGTACCTGAACCTGTACAAAATCGACATCCCGAAAAAAATCAAACGTCTGTACTTCTACAACCCGGA
CATGGAACCGAAACTGTTCGCTCGTAACCTGTCTCGTGTTAACAACTTCAAATTCCAGGATTCTAACG
ACCTGGTATGGATAGAAATCCCGGACATCGACTTCCAGATCACCCCGAAAAACGTTTTCCAGTACAAA
GTTGAAAAAGAAGAAATCATCAAAGAAGAAGAAGACAAAAAACTGTTCGTTAAAACCCTGTACAAATA
CATCAAAAAACTGTTCCTGGACAACGACTTCTACTTCAAAAAAGGTAACAACTTCATCTCTAACTCTG

AAGTTTTCTCTCTGGACTCTAACGAAAACGTTAACGCTCACCTGACCTACAAAATCAAAATCCACAAC
ATCTCTAACGAATACTACCTGTCTATCCTGCCGAAATTCACCTTCCTGTCTAAAGAACCGGCTCTGGA
ATCTGCTATCAAATCTGGTTACCTGTACAACATCAAATCTGGTAAATCTTTCCCGTACATCTCTGGTC
TGGACGGTATCCTGAAAATCGACATCGGTAACAACCAGATCGTTGAAGTTGCTTACCCGGAAAACTAC
CTGTTCAACTTCACCACCCGTGACGCTGAAAAATACGGTTTCTCTAAAGAAGTTCACGAAATCTACAA
AAACAAAGTATTCGAAGGCTTCAAAAAAATCCCAAAAACTCTCGGCTTCCTGAACAAAATAACCAACC
TGAACGAGAACTACCAGCTGAAAGACGGTTACAAAATCTTCATCAACGTTATCTACAAATTCAAAAAC
GGTGAATCTCGTTACGCTAAAGACGTTTTCAAATACTCTTTCTACAAAAACGAACAGCCGCTGAAAGC
TATCTTCTTCTTCTCTTCTAAAAAACAGTTCTTCGAAGTTCAGAAATCTCTGAAAGAACTGTTCCACA
ACAAACACTCTGTTTTCTACCGTGCTGCTGCTGAACTGGGTTTCTCTAAAGTTGAATTCCTGCGTGAC
TCTAAAACCAAATCTTCTGCTTTCCTGTACAACCCGGAAGAATTCACCGTTAAAAACACCGAATTCAT
CAACCAGATCGAAGACAACGTTATGGCTATCGTTCTGCTGGACAAATACATCGGTAACATCGACCCGC
TGGTTCGTAACTTCCCGGACAACCTGATCCTGCAGCCGATCCTGAAAGAAAAACTGGAAGACATCAAA
CCGTTCATCATCAAATCTTACGTTTACAAAATGGGTAACTTCATCCCGGAATGCAAACCGTTCATCCT
GAAAAAAATGGAAGACAAAGAAAAAAACCTGTACATCGGTATCGACCTGTCTCACGACACCTACGCTC
GTAAAACCAACCTGTGCATCGCTGCTGTTGACAACACCGGTGACATCCTTTATATCGGTAAACACAAA
AACCTGGAACTGAACGAAAAAATGAACCTTGATATCCTCGAAAAAGAATACATCAAAGCTTTCGAAAA
ATACATCGAAAAATTCAACGTTTCTCCAGAGAACGTCTTCATCCTGCGTGACGGTAGGTTCATCGAAG
ACATAGAAATCATCAAAAACTTCATCTCTTACAACGACACCAAATACACCCTGGTTGAAGTTAACAAA
AACACCAACATCAACTCTTACGACGACCTGAAAGAATGGATCATCAAACTGGACGAAAACACCTACAT
CTACTACCCGAAAACCTTCCTGAACCAGAAAGGTGTTGAAGTTAAAATCCTGGAAAACAACACCGACT
ACACCATCGAAGAAATCATCGAACAGATCTACCTGCTGACCCGTGTTGCTCACTCTACCCCGTACACC
AACTACAAACTGCCGTACCCGCTGCACATCGCTAACAAAGTTGCTCTGACCGACTACGAATGGAAACT
CTATATCCCGTACTAA

MpIHF (codon optimized)

ATGAACAAAAAAGAACTGGTTTCTGCTCTGGCTGAAAAACTGTCTGTTACCAAAAAAGAAGCTGCTCT
GTTCGTTGACTCTTTCGTTGACGTTGTTTCTGAAGCTCTGTCTAAAGGTGACAACGTTAAAATCGTTG
GTTTCGGTACCTTCGAAGTTGTTGAACGTAAACCGCGTAAAGGTGTTAACCCGCAGACCAAAGAAACC
ATCGAAATCCCGGGTGGTAAAGTTCCGAAATTCAAAGCTGGTAAAGAACTGAAAGCTAAAGTTAAATA
ATAA

DbCAPP (codon optimized

ATGATTCAGGTTGGTCGTAGCATTACCACCGTTGGTGATCCGCTGCGTAAAATGGAAGTTGAGCAGCT
GTTCCAGGTGATTAAAAGCCCGAGTCCGGAACTGCAGAACAAAATTCGTCAGCTGCGTATTGTGCGTA
ACATTGATGCAAAACAGTATGCAGCACTGAAAAAACAGCTGCCGTATTTTGTGTGCGGTATCTTTAAT
CCGAACATTCGTCGTACCGAAAACTTTGCCTATTGCGATTGCTTTGTGATCGACATTGATCACATTAG
CGAAAAAGGTCTGAATGTTCAGAGCCTGCGTCAGAAAATTGAAAATGATAGTCGTACCCTGCTGAGCT
TTGTTAGTCCGGGTGAAGATGGTATTAAAGTGCTGTTTCGTCTGGCAGAACGTTGTTATGATGCAGGT
ATTTTTACCGCCTTCTACAAAAGCTTTCTGACCGATTTTAGCCGTCAGTATGGTCTGCAGCAGGTTGC
CGATCTGCGTACCAGTGATGTTACCCGTGCATGTTTTGTTAGCTTTGATCCGAATGCCTTTTATAACC
CGAATGCAAGCCCGATTGAAATCCATGCATTTATCGATGTGAACAACACCTTTGAAACCCTGCAGGCA
AAAAAAAGCATTGAGAACGAACTGAAAAAGCAACAGCGTCAGCCGGAACCGCTGGTTGAAAAAAGTGA
TGTTGATGATGATGTGATCGGCAAAATCAAAGCCATCCTGTTTAAAGTTCCGAAGCCGATCGAAAAAC
CGCCTGCATATGTTCCAGAACAGCTGAATGAAATTATGACCGATCTGCAGGTTTTTCTGCTGGATGCC
GGTGTTGTTGTTAATGAAATTCGCAGCATTAGCTACGGCAAAAAGATTCAGGCAAGCATTGCACATAA

ACAGGCCGAAGTTAACCTGTTTTATGGTAAACGTGGTTTTAGCGTTGTGCAGAGCAGCCGTACCGGCA
CCAGCGCAGAAATGAATCAGCTGCTGGCAGATCTGGTTCAGGCATTTATTCAGAGCCGT

DbCas1-DbCas2 (codon optimized)

CACCACAGCCAGGATCTGGAAGTTCTGTTTCAGGGTCCGATGGATCTGATGATTAATACCTTTGGCAC
CAGCCTGAGCCGTGATAATGATTGTTTTGTGATCCTGCACAAAGATGGTAAACAGCGTGTTCCGGTTG
AAGGTATTACCAGCATTCAGATTGGTCGTGGTGCACAGATTACCAGTGATGCAGTTCTGCTGGCAATT
CAGAACGAAATTGAAATCCTGTTTCTGGATAATAGCGGTGAACCGGTTGGTCGTGTTTGGAGCAACAA
ATATGGTAGCATTAGCACCATTCGTAAAGGCCAGCTGAATTTCACCCTGAGCAAAGATGCAGTTAGCT
GGATTAAAGAAGTGATCGCACAGAAAATGGAAAATCAGCAGGCACTGATTCTGAGCATGATGGTTAAT
GATGATCGTACCCAGCATCTGGTTGATAAAGCAATTCAGCGCATTGAAGATTATCGTGCCAAAGTTAA
AAGCCTGGATGGTGAAGTTATTAGCGATATTGCACCGGCACTGCGTGGTTGGGAAGGTCAGGCAAGCC
GTATCTATTTTGAAACCCTGAACCTGTTCATTCCGGATAAACTGCGTTTTGCACAGCGTAGCCAGCAT
CCGGCAACCGATGTTGTTAATGCATTTCTGAATTATGGCTACGGCTTCCTGTATGGTAAAATTGAAGG
TGCCCTGATTCGTGCAGGTATTGATCCGTATATTGGTGTGTTTCACCGCGACGATTATAATCGCCCTG
TTCTGGTTTATGATGTGATTGAACTGTATCGCATCTGGGTCGATTATGTTGTTTTTACCCTGGTTATG
CAGCGCGATATCATTACCGATGAATTCTATAGCGTGAAATCCGATGGTAGCTATTGGCTGGAAGCACT
GGGTCGTCGTATTGTTATTCAGAGCCTGAATGATTATCTGGATGAAGTGATTCTGATGAACGGCATTA
ATCGTAGCCGTCTGAGCCATATTTTTCTGTATGCACAGAATCTGGCCCAGAAATTCAAAACCTATATG
TAATAATGCTTAAGTCGAACAGAAAGTAATCGTATTGTACACGGCCGCATAATCGAAATTAATACGAC
TCACTATAGGGGAATTGTGAGCGGATAACAATTCCCCATCTTAGTATATTAGTTAAGTATAAGAAGGA
GATATACATATGTGGTCCCATCCGCAGTTTGAAAAACTGGAAGTTTTATTTCAAGGCCCTATGAAAAA
ACGTCAGCCGCTGAGCTTTGTTGAGAAAATGAAAAAATTCTCACAGGCAGGTATGAGCCGTCCGCTGA
GCCCGAATCGTCATGTGGGTAATCTGGAAGGCCTGAAATCACTGGATGAACGTGTTGCCTTTCTGCTG
AATATTGTTAAACAGCCGAAACGTCCGGCAAACAACATGCTGTTTTTGTGATGTATGATATCGAAAG
CACCCGTGTGCGTAATCAGATTGTTAAATATCTGCTGCGTAGCGGTTGTACCCGTGTTCAGAAATCAA
TTTTTCTGGCCGATCTGCCGATTGATACCTATGAACGTATTCGTAGCGAACTGGCACTGGTTCAGCAG
TGTTATGAAAATGAAGATAGCATTCTGGTTGTGCCGATTAGCAGCGATTATCTGCAGAGCATGAAAAT
CATTGGCAAAAACATTGATGTGGACCTGATTATGAATGCCCGTAATACCCTGTTTTTTGATAACCTA
GGCTGCTG

DbRecD (codon optimized)

ATGGAAAAAGAAAAAATTGTGGTTCATAAAGAAAGCGTGTTTGAACTGACCCCGAATCAGAAAAAAAC
CTTTCAGCACTTTCAGAAATTCGTGGCCGATAATGAACTGAAAGTGTTTATCCTGAAAGGCTATGCAG
GCACCGGTAAAACCACACTGATTCGCTATTTCATTGATGAAATTGCCCGTCAGGATAATGCACAGTAT
ACCCTGATGGCAAGCACCGGTCGTGCAGCAAAAATTCTGACCAATATGAGCCGTCGTAAAGCACAGAC
CATTCATAGCGTTATCTATGTGTTCAACGATTTCAACCAGGATCTGGAAGAAGTGATTCGTAACGAAG
ATGAAGTTGGTGTTGATAAAACCGGTCAGCTGTTTCTGACCTATCTGCTGACACCGGTTAAAGAGAAA
AACGATAAACAGCGCTATTACATCGTTGATGAAGCAAGCATGATTAGCGATACCATCGATGAAAATGC
AGTTCAGGCACAGTTTGGTAGCGGTCGTACCCTGAGCGATCTGATGAAATATGATCCGGATGGCAAAT
TTGTGTTTGTGGGTGATGAATGTCAGCTGCCTCCGATTGGTCAGGATCTGAGTCCGGCACTGAGCGTT
GAATATTTTCGTAATGTTCTGAATGTGCACGCCGTGGAATACACCCTGACCGATATTGTTCGTCAGCA
GCTGGATAATAGCATTATTCTGGCAGCACAGCGTATTCGTCGTCTGTGTATTGAACCGCCTCAGGTTA
AATGGGGTAAACTGCCGCTGGGTAATTATCAGCATATTCATCTGCATCATGATATCGCCAGCATGATC
AACGATTACATTAATCTGATTCGCAACCGCAATTTTGAAGCAGCAACCCTGATTAGCAGCAGCAATGC
AAAATGCAATAACCTGAACAAACTGATTCGTAGCGCACTGCATTATCGTAATACCCTGCAAGAGGGTG
ATCTGCTGATGGTTACACAGAATAATCCGATTAGTGGTCTGATGAATGGTGATATGGTTATGGTTGAA
CAGGTGAAAAAAGTGCGTTATCAGCGTGCCCAGCTGAGCTTTCTGCTGGTTGAAGTTAAAGAACTGGT
TAGTGGTCGTCGTTTTAGCCAGTATGTGATTGAAGATATTCTGTATGGTAATGCACCGAATCTGCTGC
CTGGTCAGCAAAAAGCACTGTTCATTGATTTTTACCGTCGCATGAAAGAACAGGGTGTTAAACAGAAA

AGCAGCCAGTTTCGTGAACGTCTGATGGATGATGAATTCCTGAATGCACTGCGTTGCGTTTATGGCTA
TGCAATTACCTGTCATAAAGCACAAGGTGGTGAATGGGATAGCGTTTTTGTTGATATTCCGCGTAATC
TGACCCTGGAAGCACGTAGCGCAGATTATCAGTGGATTTATACCGCAGTTACCCGTGCACGTAAACAG
CTGCATATTGTGAACGATTTCTACATTAGCAAA
