# Supplementary material for: CRISPR-Associated Primase-Polymerases are implicated in prokaryotic CRISPR-Cas adaptation
Source: Nat Commun. 2021 Jun 17;12:3690. doi: 10.1038/s41467-021-23535-9 (PMC8211822; doi:10.1038/s41467-021-23535-9)
Supplement: Supplementary file 5 — Description of Additional Supplementary Files [file 41467_2021_23535_MOESM5_ESM.docx]

Descriptions of additional supplementary information

Title: Supplementary Data 1

Description: PSI-BLAST (5 iterations) hits dataset retrieved for DbCAPP query

Title: Supplementary Data 2

Description: PSI-BLAST (5 iterations) hits dataset retrieved for MpCAPP query.

Title: Supplementary Data 3

Description: Tables of oligonucleotides and plasmids used in this study.
